# Identification of symbiotic nitrogen fixation‐modulating factors in alfalfa and mechanism elucidation of MsHHO3

**DOI:** 10.1111/tpj.70831

**Published:** 2026-03-29

**Authors:** Yajing Wu, Qian Liu, Fei He, Siqi Wang, Yuxuan Ding, Junmei Kang, Pengbo Liang, Qingchuan Yang, Xue Wang

**Affiliations:** ^1^ Institute of Animal Science The Chinese Academy of Agricultural Sciences Beijing 100193 China; ^2^ State Key Laboratory of Plant Environmental Resilience, Frontiers Science Center for Molecular Design Breeding (MOE), College of Biological Sciences China Agricultural University Beijing 100193 China; ^3^ MOA Key Laboratory of Soil Microbiology, and Rhizobium Research Center China Agricultural University Beijing 100193 China

**Keywords:** nitrate, nodule, HHO3, MYC2, alfalfa

## Abstract

Symbiotic nitrogen fixation (SNF), a unique nitrogen acquisition mechanism formed through the interaction between leguminous plants and rhizobia, plays a critical role in reducing dependence on chemical fertilizers. However, in alfalfa (*Medicago sativa* L.), the mechanisms underlying nitrate‐responsive SNF remain poorly understood. In this study, we elucidate a nitrate‐responsive regulatory network governing SNF in alfalfa and identify MsHHO3 as a key regulator. qRT‐PCR analysis revealed that *MsHHO3* expression is significantly higher in the LAPIOSZELEI (LA) variety, which exhibits fewer nodules and lower nitrogenase activity, than in the TING SI (TS) variety, which shows a greater number of nodules and higher nitrogenase activity. Morphological characterization demonstrated that *MsHHO3*‐overexpressing lines exhibited significantly reduced nodule number, nodule fresh weight, and nitrogenase activity, whereas suppression of *MsHHO3* by RNA interference (RNAi) in alfalfa resulted in an opposite phenotype. The CRISPR/Cas9 mutant of *MtHHO3*, a *Medicago truncatula* homolog of *MsHHO3*, displayed the similar phenotype as *MsHHO3*‐Ri. RNA‐seq, ChIP‐seq, and qRT‐PCR analysis showed that a set of nodulation‐associated genes were altered in *MsHHO3*‐overexpressing plants, as well as in *MsHHO3*‐Ri and *mthho3* mutants. Among these genes, several hormone‐related TF‐encoding genes were directly regulated, including the JA signaling pathway master gene *MsMYC2*. EMSA and dual‐luciferase reporter assay further demonstrated that MsHHO3 can directly bind to the *MsMYC2* promoter. We propose that MsHHO3 regulates SNF by modulating *MsMYC2* and other intermediate TFs, orchestrating a transcriptional cascade that ensures precise fine‐tuning of the nodulation process. These findings provide novel mechanistic insights into nitrate‐responsive regulation of SNF in alfalfa.

## Significance Statement

HHO3 acts as a key regulator of nitrate‐responsive SNF by directly targeting several intermediate hormone‐related TF‐encoding genes (*MsMYC2*, *Ms* *RAV1*, *Ms* *bHLH18*, *Ms* *MYB108*, *Ms* *MYB11*) to initiate a transcriptional cascade, which in turn coordinates the expression of core nodulation genes *YUC*, *PIN*, and *LBD* for precise regulation.

## INTRODUCTION

Alfalfa (*Medicago sativa* L.) is an important perennial leguminous forage that is cultivated worldwide for its high nutritional quality, strong adaptability and high yield (Radovic et al., 2009). It is rich in protein, vitamins, and minerals, making it a primary nutritional source for livestock. Additionally, alfalfa cultivation is important for ecosystem and soil improvement (Wang & Zhang, 2023). As a legume, alfalfa has a symbiotic relationship with rhizobia, forming nodules that enable gaseous nitrogen (N_2_)‐fixation through symbiotic nitrogen fixation (SNF) (Radovic et al., 2009). This process helps improve agricultural productivity by providing plants with N, enriches the soil, and reduces the need for N fertilizers while supplying N for subsequent crops (Gao et al., 2022). However, to maintain crop growth and obtain high yields, N fertilizer is often overused. Elevated N levels affect all stages of SNF, resulting in reduced nodule numbers, impaired nitrogenase activity, and accelerated nodule senescence (Qiao et al., 2023; Wang et al., 2023; Wang, Huang, et al., 2020). Nodule senescence is accompanied by leghemoglobin degradation (indicated by a color change from pink to green in nodules), elevated reactive oxygen species (ROS) levels, symbiosome degradation, and loss of nitrogenase activity (Dupont et al., 2012; Kazmierczak et al., 2020). This consequently leads to the irreversible loss of N‐fixing function in nodules, which severely impairs SNF efficiency (Dupont et al., 2012). Therefore, exploring the molecular mechanisms of alfalfa nodulation can help breed varieties with high N_2_ fixation ability and reduce fertilizer input (Yan & Bisseling, 2024).

The N concentration in soil changes rapidly, usually ranging from micromolar to millimolar levels within a few meters (Miller et al., 2007). Nodulation is an energy‐consuming process and relies on the available nitrate concentration. Therefore, to cope with fluctuating rhizosphere N conditions and tightly regulate N acquisition, legume plants have evolved intricate mechanisms to regulate nodule organogenesis, nodule number, and nitrogenase activity (Lin et al., 2024; Qiao et al., 2024; Wang, Huang, et al., 2020; Zhu et al., 2020).

Transcription factors (TFs) play essential regulatory roles during legume nodulation (Qiao et al., 2023; Schiessl & Jhu, 2025; Wang et al., 2023). Among them, NODULE INCEPTION (NIN) has been reported to be a hub gene for symbiotic infection (Lee et al., 2024; Schiessl et al., 2019; Schiessl & Jhu, 2025). The NIN‐like proteins (NLPs) are key regulators in response to nitrate (Konishi & Yanagisawa, 2013; Lee et al., 2024; Lin et al., 2018; Zhong et al., 2023). Recent reports showed that the NLPs also play important roles in the nodules of leguminous plants. NLP1 and NLP4 inhibit nodule initiation by activating CLE peptides in *Medicago truncatula* (*M. truncatula*) and soybean (Fu et al., 2024). NLP1 modulates the nodule number of *M. truncatula* by regulating nitrate transporter gene *NRT2.1* under varying nitrate conditions (Luo et al., 2023). The basic helix–loop–helix (bHLH) TFs play essential roles in jasmonate (JA) signaling pathway and plant developmental process (Goossens et al., 2017). MYC2, a member of the bHLH family, is a key regulator in JA signaling and integrates different signals to regulate plant growth (Kazan & Manners, 2013). In *M. truncatula*, MYC2 has been reported to mediate root nodule symbiosis. The nodule number, weight, and nitrogenase activity are severely inhibited in *myc2* mutant (Guo et al., 2024). In soybean, overexpression of *GmbHLH93* leads to higher nodule numbers (Sun et al., 2024). These results indicate that *bHLH* TFs and the regulation of JA signaling pathway are essential for nodulation. In addition, other hormone signaling pathways and their regulation are also critical for the nodulation of leguminous plants, such as auxin, ethylene, gibberellin (GA), and brassinosteroid (BR), etc. (Breakspear et al., 2014; Drapek et al., 2024; Lin et al., 2020; Schiessl et al., 2019). Exploring the underlying mechanisms of plant hormone signaling is important for improving SNF.

The NITRATE‐INDUCED GARP‐TYPE TRANSCRIPTIONAL REPRESSOR 1 (NIGT1) was initially identified in rice as a hub TF in the plant N signaling network (Sawaki et al., 2013; Ueda et al., 2020). Four NIGT1 homologs have been reported to negatively regulate the response of Arabidopsis to N starvation by directly repressing the transcription of *NRT2.1* and *NRT2.4* (Kiba et al., 2018; Maeda et al., 2018). The rice mutant *hho3* (*nigt1.1*) significantly enhances rice N use efficiency and yield (Liu et al., 2023). More recently, soybean GmNRAMP2s (Natural Resistance‐Associated Macrophage Protein 2a and 2b) were reported to regulate SNF efficiency by affecting iron (Fe) homeostasis (Zhou et al., 2024). These two genes can be regulated by GmNIGT1a and GmNIGT1b, revealing a link between N signaling and Fe transport in soybean nodules (Zhou et al., 2024).

In this study, we report that *MsHHO3* encodes the alfalfa ortholog of the Arabidopsis N signaling gene *NIGT1.1*. *MsHHO3* is primarily expressed in root systems and is induced by nitrate. MsHHO3 plays a crucial role in modulating SNF, as further verified in alfalfa varieties with varying nodule numbers and nitrogenase activity. Chromatin immunoprecipitation followed by sequencing (ChIP‐seq), RNA sequencing (RNA‐seq), and luciferase assays were employed to identify downstream genes regulated by MsHHO3. The results indicate that MsHHO3 negatively modulates SNF through regulating several pathways, particularly the genes involved in phytohormone signaling.

## RESULTS

### High nitrate inhibits alfalfa SNF

**Figure 1:**
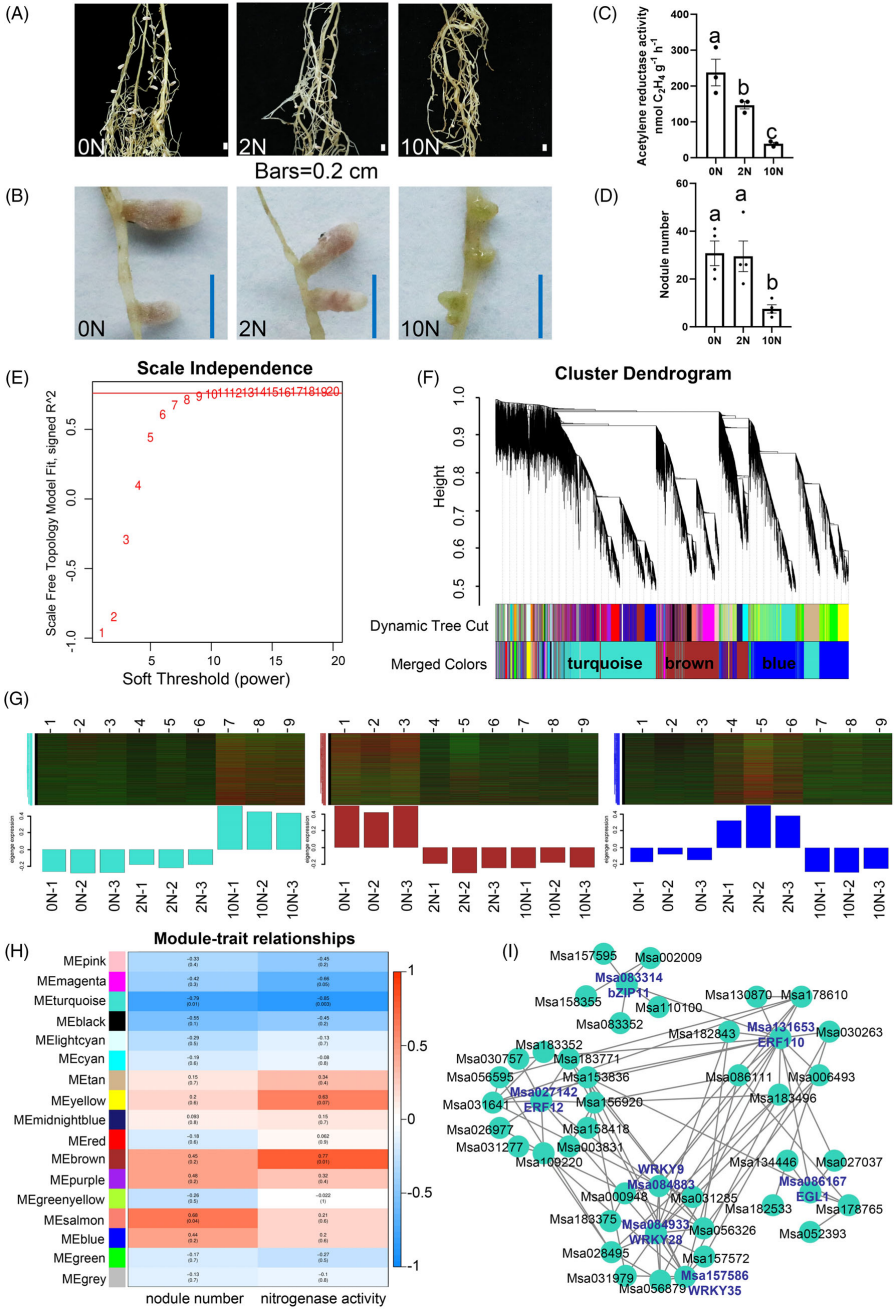
Weighted co‐expression network analysis. (A, B) Nodulation comparison of alfalfa Zhongmu No. 4 variety under different nitrate conditions. Scale bar = 0.2 cm. (C) Nitrogenase activity. (D) Total nodule number. (E) Soft thresholding visualization plot. The correlation coefficient is 0.8 and threshold is 9. (F) Module hierarchical clustering dendrogram of DEGs. (G) Top 3 module eigengene expression profiles, including turquoise, brown, and blue modules. Eigengene expression level >0 indicates upregulation; <0 indicates downregulation. (H) Correlation analysis between modules and number of nodules, as well as nitrogenase activity. (I) Transcription factors regulatory network analysis in turquoise module. One‐way analysis of variance (ANOVA) was used to identify significant differences, which were denoted by letters (*P* < 0.05).

The N concentration in the soil solution is highly variable (Miller et al., 2007). High N levels inhibit SNF, including nodule formation, nodule development, nitrogenase activity, and accelerate nodule senescence (Dupont et al., 2012; Qiao et al., 2023; Wang et al., 2023). To investigate the nodulation of alfalfa under different nitrate concentrations, seedlings of the alfalfa cultivar Zhongmu No. 4, which has a fully assembled and well‐annotated genome (Long et al., 2022), were treated with 0 mM (0N), 2 mM (2N), and 10 mM KNO_3_ (10N), respectively. The results showed that, when treated with 0N or 2N, alfalfa roots produced pink N‐fixing nodules. When subjected to 10N, nodules appeared white, with some even turning green, exhibiting a senescence phenotype (Figure 1A,B). Consistently, nitrogenase activity in these nodules was significantly lower than that in the 0N or 2N treatments (Figure 1C). Meanwhile, the nodule number formed on roots under 10N condition was also markedly decreased (Figure 1D). To explore the genome‐wide differentially expressed genes (DEGs) involved in the regulation of alfalfa SNF, RNA‐Seq was conducted using nodules from roots treated with different KNO_3_ concentrations. The DEGs between 0N and 10N were further analyzed using Kyoto Encyclopedia of Genes and Genomes (KEGG) analysis. The results showed that the top 20 enriched pathways involved phenylpropanoid biosynthesis, plant hormone signal transduction, plant–pathogen interaction, glutathione metabolism, ABC transporters, flavonoid biosynthesis, etc. (Figure S1). These results suggest that these pathways play a role in the regulation of alfalfa nodulation.

### Co‐expression analysis of alfalfa under different nitrate conditions

Transcriptional regulation plays a pivotal role in the SNF process (Qiao et al., 2023). To identify TFs that control alfalfa SNF in response to different N levels, we conducted weighted gene co‐expression network analysis (WGCNA) using 25 216 expressed genes, incorporating data on nodule numbers and nitrogenase activity from plants treated with 0N, 2N, and 10N. Genes were clustered into 17 modules based on similar expression patterns (Figure 1E,F). Among these modules, the turquoise, brown, and blue modules contained the largest number of co‐expressed genes. We subsequently conducted Gene Ontology (GO) enrichment analysis on DEGs from these three modules to dissect their functional divergence. The analysis revealed that genes in each module are associated with distinct biological processes, and the turquoise module was found to be enriched in genes associated with sequence‐specific DNA binding (Figure S2). The impacts of different nitrate treatments on gene expression for these three modules were analyzed, and eigengenes were used to illustrate the overall gene expression patterns. The results demonstrated that within the turquoise module, the transcription levels of eigengenes were downregulated under both 0N and 2N, but upregulated under 10N treatment (Figure 1G), suggesting that gene expression in this module is repressed by nitrate deficiency and induced by high nitrate level. In contrast, gene expression in the brown module was downregulated by both 2N and 10N treatments, while gene expression in the blue module was only induced by 2N treatment. For the turquoise module, gene expression patterns under 0N and 2N exhibited congruent trends, which is consistent with observed nodulation phenotypes (Figure 1G). Furthermore, correlation analysis between modules and phenotypic traits revealed that the turquoise module exhibited the strongest correlation with nodule number and nitrogenase activity (Figure 1H). Based on these results, we focused on the turquoise module to further investigate how high N affects SNF.

**Figure 2:**
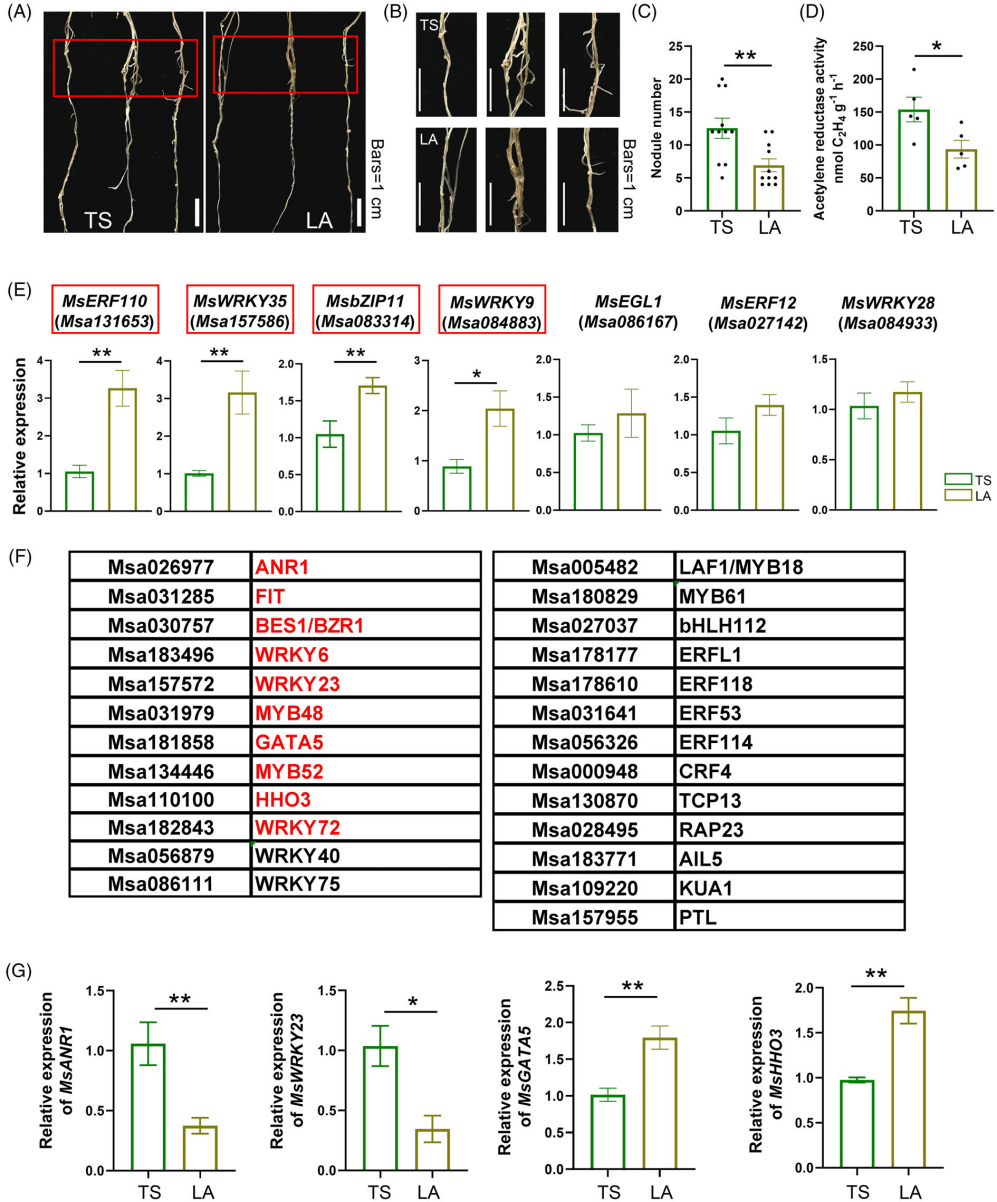
Analysis of hub TF‐encoding genes in TING SI (TS) and LAPIOSZEI (LA) alfalfa varieties. (A) Nodulation phenotypes of TS and LA varieties under N‐deficient conditions at 14 days postinoculation (dpi). Scale bars = 1 cm. (B) Magnified images of the red rectangular boxes in (A), bars = 1 cm. (C) Total nodule number. (D) Nitrogenase activity. (E) Expression analysis of highly connected genes in TS and LA alfalfa varieties. Genes highlighted by red boxes indicate those with significant differences. (F) TFs interact with ERF110, WRKY35, and bZIP11. Red‐highlighted TFs are involved in N signaling or nodulation. (G) Expression analysis of red‐highlighted significantly different genes between TS and LA alfalfa varieties. Two‐tailed student's *t*‐test was used to identify significant differences (*P < 0.05, **P < 0.01).

Module membership value (kME) revealed that there were 2863 genes with |kME| ≥ 0.85 in the turquoise module, and 82 TF‐encoding genes were identified. The TFs were divided into five clusters based on k‐means analysis using STRING version 12.0. Within the clusters, TFs Msa027142 (ERF12), Msa131653 (ERF110), Msa084933 (WRKY28), Msa083314 (bZIP11/ATB2), and Msa086167 (EGL1) exhibited higher connectivity. Aside from them, two TFs, Msa157586 (WRKY35) and Msa084883 (WRKY9), also had high connectivity according to network analysis in Cytoscape (Figure 1I). Among these genes, *bZIP11/ATB2* is a well‐known marker gene for nodule senescence and has been reported to play an important role in nodule formation (D'haeseleer et al., 2010; Van Dingenen et al., 2023). In *M. truncatula*, the *MtATB2*‐overexpressing lines exhibit lower nodule numbers. To determine whether these TFs participate in alfalfa SNF, gene expression levels were measured in two alfalfa varieties, TING SI (TS) and LAPIOSZELEI (LA). Under N‐deficient conditions, TS produced more nodules and exhibited higher nitrogenase activity than LA (Figure 2A–D; Figure S3). Under high N conditions, no significant genotypic differences were observed (Figure S4), suggesting that TS and LA respond differently to N availability. Accordingly, gene expression analysis was performed under N‐deficient conditions. The results showed that the expression levels of *ERF110*, *WRKY35*, *bZIP11/ATB2*, and *WRKY9* were significantly higher in LA than in TS, indicating that these genes may play negative roles in modulating alfalfa SNF (Figure 2E).

The interacting genes of above TFs were also analyzed and listed in Figure 2(F). Several TFs were found to be involved in N signaling or nodulation, including *ANR1* (Castaings et al., 2009; Wang, Zhang, et al., 2020), *FIT* (FER‐LIKE IRON DEFICIENCY‐INDUCED TRANSCRIPTION FACTOR) (Luo et al., 2012), *BES1* (Chen et al., 2023), *WRKY6* (Wang et al., 2023), *WRKY23* (Alvarez et al., 2020), *MYB48* (Gaudinier et al., 2018), *GATA5* (Liu et al., 2019), *MYB52* (Ueda et al., 2020), *HHO3* (Ueda et al., 2020), and *WRKY72* (Sun et al., 2024) (Figure 2F). Among these, *ANR1*, *WRKY23*, *GATA5*, and *HHO3* were differently expressed between TS and LA (Figure 2G; Figure S5). HHO3 is well established as a key regulator of nitrate absorption and metabolism (Kiba et al., 2018; Maeda et al., 2018; Yang et al., 2024). The homologs of bZIP11 and HHO3 in rice were also reported to interact with each other, and both of them play key roles in rice N utilization (Ueda et al., 2020). The *oshho3* single mutant significantly enhanced rice N use efficiency (NUE) and yield (Liu et al., 2023). Based on these results, MsHHO3 (Msa110100) was predicted to play a role in regulating alfalfa SNF and was selected for further functional analysis.

### Expression pattern of *HHO3*

**Figure 3:**
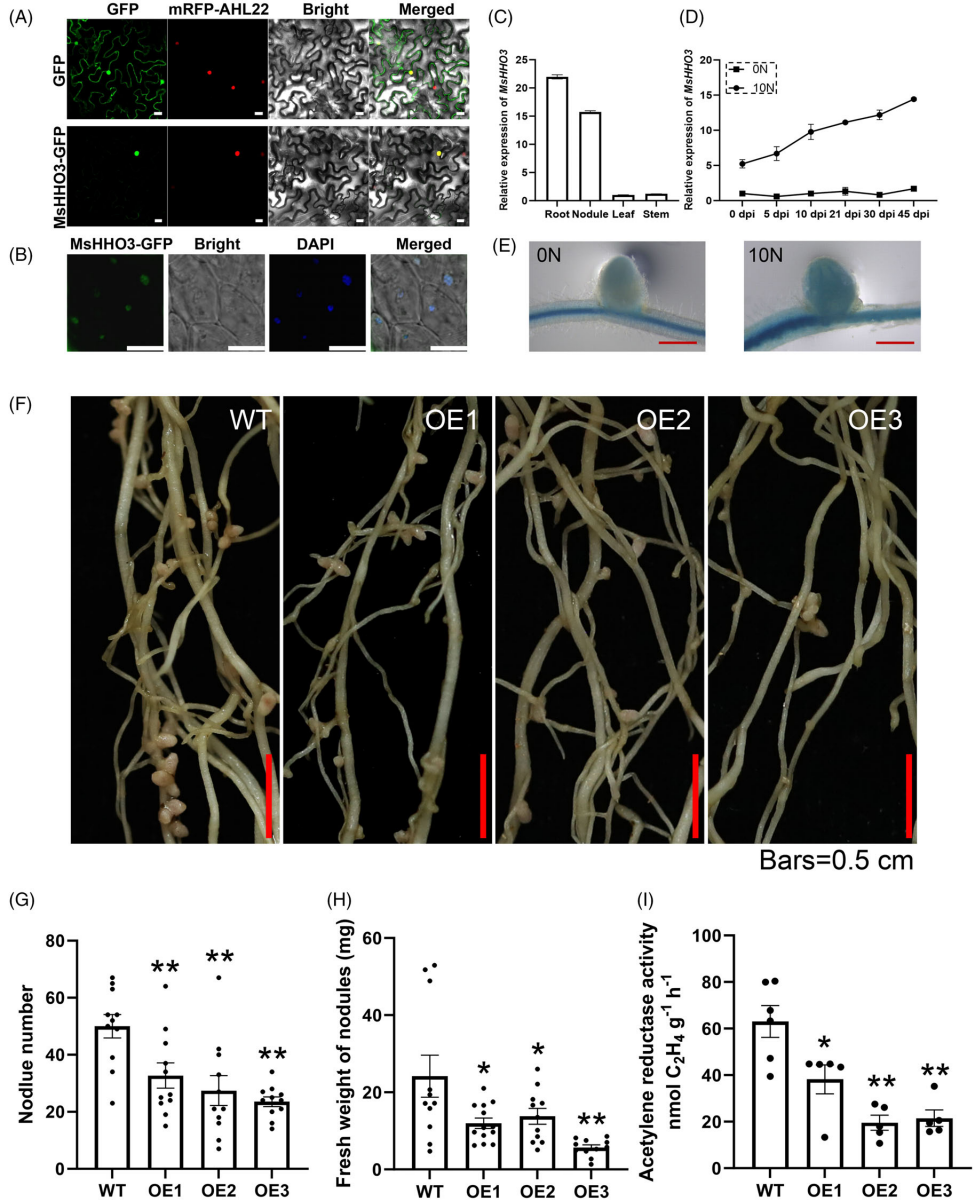
MsHHO3 negatively regulates SNF in alfalfa. (A, B) Subcellular localization of MsHHO3 in *Nicotiana benthamiana* leaves and nodule cells. Scale bar = 20 μm. (C) Expression levels of *MsHHO3* in different tissues of alfalfa. (D) Expression analysis of *MsHHO3* in alfalfa roots at different days post inoculation (dpi). (E) GUS staining analysis of *ProMtHHO3:GUS* transgenic plants under different nitrate treatments. Scale bar = 20 μm. (F) Phenotypic comparison of *MsHHO3*‐overexpressing alfalfa lines and wild‐type (Zhongmu No. 4). Scale bar = 0.5 cm. (G) Total nodule number. (H) Fresh weight (FW) of nodules. (I) Nitrogenase activity. Plants grown without nitrogen for 7 days were inoculated with rhizobia, and nodule phenotypes were observed and calculated at 14 dpi. Two‐tailed Student's *t*‐test was used to identify significant differences (*P < 0.05, **P < 0.01).

HHO3, also named NIGT1.1, belongs to the NIGT1/HRS1/HHO subfamily of the GARP superfamily. Amino acid sequences alignment showed that MsHHO3 has 96.42% identity with *M. truncatula* HHO3 and 43.42% identity with Arabidopsis HHO3 (Figure S6A–C). The coding sequence (CDS) of *MsHHO3* was amplified from the Zhongmu No. 4 variety. To analyze the subcellular localization of MsHHO3, a *Super:MsHHO3‐GFP* fusion vector was constructed. The construct was transformed into *Nicotiana benthamiana* leaves and alfalfa, respectively. The results showed that MsHHO3‐GFP accumulates in the nucleus, and this subcellular localization was not altered by different N treatments (Figure 3A,B; Figure S6D,E). The expression of *MsHHO3* in four distinct tissues of alfalfa was detected via quantitative real‐time PCR (qRT‐PCR). The results showed that *MsHHO3* expression was relatively high in roots and nodules, suggesting that MsHHO3 might play a role in lateral organs (Figure 3C). qRT‐PCR analysis revealed that the expression of *MsHHO3* was significantly induced by high N level. Specifically, *MsHHO3* expression exhibited a gradual upward trend from 5 days postinoculation (dpi) to 45 dpi under 10N conditions, but stayed at a low basal level with minimal fluctuation under 0N conditions (Figure 3D). To further validate the transcription of *HHO3*, the promoter of *M. truncatula HHO3* was amplified and fused with *GUS*. Staining of the stable *ProMtHHO3‐GUS* line showed higher GUS activity in roots and nodules under N‐sufficient condition (Figure 3E). In nodules, GUS activity was detected throughout the whole tissue, with the highest activity observed in vascular tissues, and staining intensity gradually increased from the nodule apex toward the base. Under N‐deficient conditions, *MsHHO3* was mainly expressed in the meristematic zone, infection zone, and transition zone of nodules (Figure S6F).

### MsHHO3 negatively regulates SNF in alfalfa

Given that *MsHHO3* is differentially expressed under varied N conditions, we constructed *MsHHO3*‐overexpressing alfalfa lines by introducing the *Super:MsHHO3‐GFP* construct into Zhongmu No. 4, which has high genetic transformation efficiency, to investigate its functional role. Three stable transgenic lines (designated as OE1, OE2, and OE3) were selected for subsequent experiments. qRT‐PCR analysis showed that the expression levels of *MsHHO3* were significantly elevated in the transgenic lines compared with the wild‐type (WT, Zhongmu No. 4) (Figure S7A). Uniform plants were vegetatively propagated via stem node cuttings from both WT and transgenic lines for phenotypic comparison. Nodulation assays were conducted, and nodule number, nodule fresh weight, and nitrogenase activity were measured (Figure 3F). The results revealed that the *MsHHO3*‐overexpressing lines exhibited significantly fewer nodules, lower total nodule fresh weight, and reduced nitrogenase activity relative to WT (Figure 3G–I; Figure S7B). These findings suggest that MsHHO3 negatively regulates SNF in alfalfa.

### Knockdown of *HHO3* expression promotes SNF

**Figure 4:**
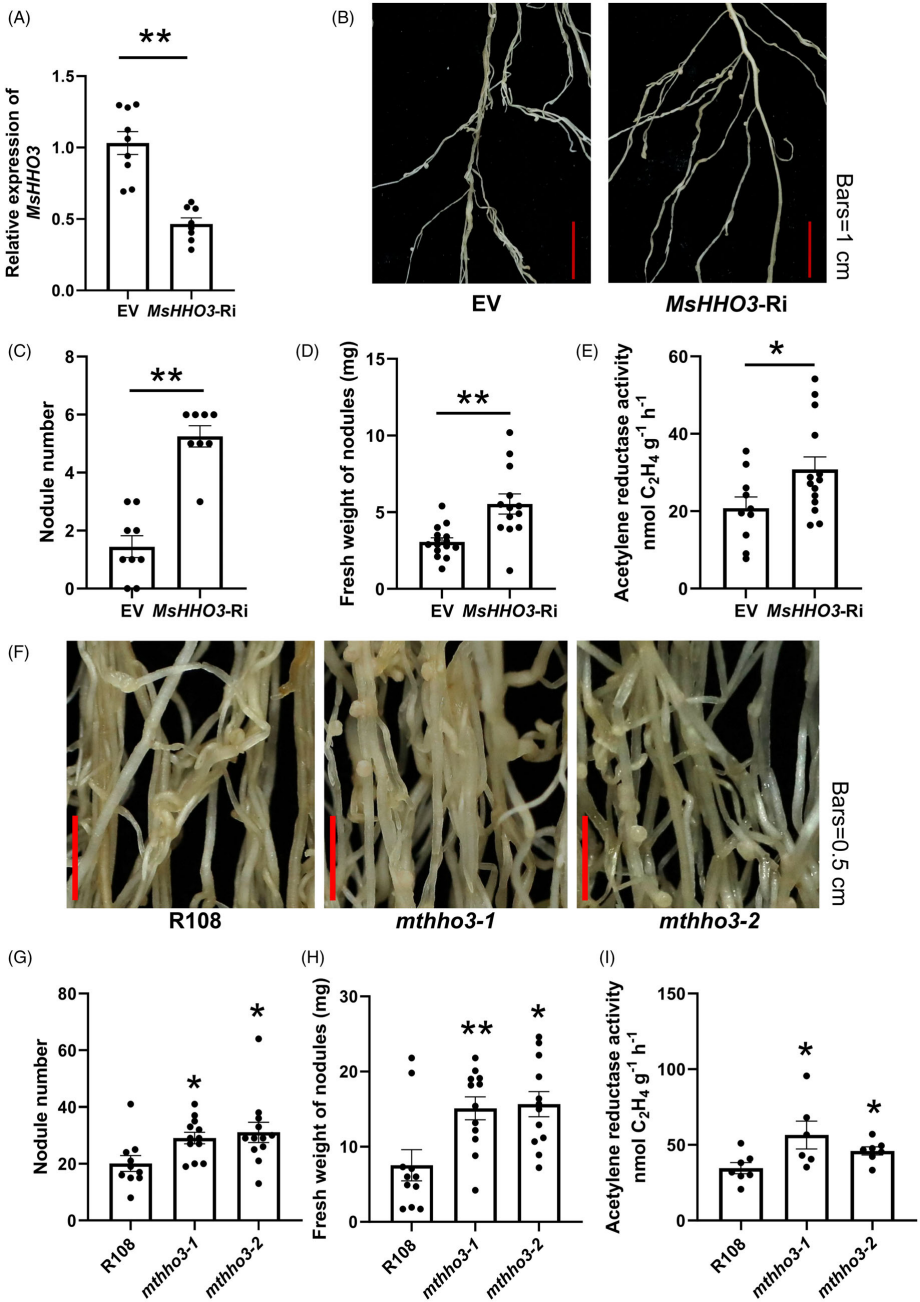
Reducing the expression or disruption of *HHO3* enhances SNF in Medicago species. (A) qRT‐PCR analysis of *MsHHO3* expression levels in hairy roots carrying the empty vector (EV) and *MsHHO3*‐Ri vector. (B) Phenotypic comparison of *MsHHO3*‐Ri and the empty vector control. (C) Total nodule number. (D) Fresh weight (FW) of nodules. (E) Nitrogenase activity. (F) Nodule phenotypes of *mthho3* mutants and wild‐type (R108). Scale bar = 0.5 cm. (G) Total nodule number. (H) FW of nodules. (I) Nitrogenase activity. Seedlings grown in high N condition for 7 days were inoculated with rhizobia, and nodule phenotypes were observed and calculated at 21 dpi. Two‐tailed Student's *t*‐test was used to identify significant differences (*P < 0.05, **P < 0.01).

To further elucidate the role of MsHHO3 in SNF, we performed RNA interference (RNAi) to knockdown *MsHHO3* expression in alfalfa hairy roots. qRT‐PCR confirmed significantly reduced *MsHHO3* expression in *MsHHO3*‐Ri roots (Figure 4A). Given that high N levels suppress SNF and *MsHHO3* is induced by elevated nitrate content, nodulation assays were conducted on *MsHHO3*‐Ri under high N conditions (Figure 4B). Uniform seedlings of *MsHHO3*‐Ri and empty vector control were used for phenotypic analysis. The *MsHHO3*‐Ri plants exhibited a significantly higher total nodule number, nodule fresh weight, and nitrogenase activity compared to the empty vector control (Figure 4C–E; Figure S8). Furthermore, there was no significant difference in nodule number, nodule fresh weight, and nitrogenase activity between *MsHHO3*‐Ri plants and the empty vector control under N‐deficient conditions (Figure S9A–F). These results demonstrate that MsHHO3 functions as a negative regulator of SNF in alfalfa under high N conditions.

To validate the functional conservation of *HHO3* within Medicago species, we targeted MtHHO3, a *M. truncatula* homolog of MsHHO3 sharing 96.14% amino acid identity, for functional analysis (Figure S6B). Using the CRISPR/Cas9 system, we designed a 20‐bp sgRNA to edit *MtHHO3* and obtained two homozygous mutant lines, *mthho3‐1* and *mthho3‐2*, which harbor a T or C nucleotide insertion at the target site, respectively (Figure S10A,B). Consistent with the approach used for the *MsHHO3*‐Ri lines, the *mthho3* mutants were also subjected to nodulation assays under high N conditions (Figure 4F). Total nodule number, nodule fresh weight, and nitrogenase activity were also significantly higher in *mthho3* mutants compared to R108 (Figure 4G–I; Figure S11). Under N‐deficient conditions, no significant differences were observed in nodule number, nodule fresh weight, and nitrogenase activity between the *mthho3* mutants and R108 (Figure S12A–D). Collectively, the consistent phenotypes of *MsHHO3* knockdown alfalfa and *MtHHO3* CRISPR/Cas9 mutant, both differing significantly from *MsHHO3*‐overexpression lines, reinforce that HHO3 functions as a negative regulator of SNF across Medicago species.

### Identify the downstream targets of MsHHO3 for SNF

**Figure 5:**
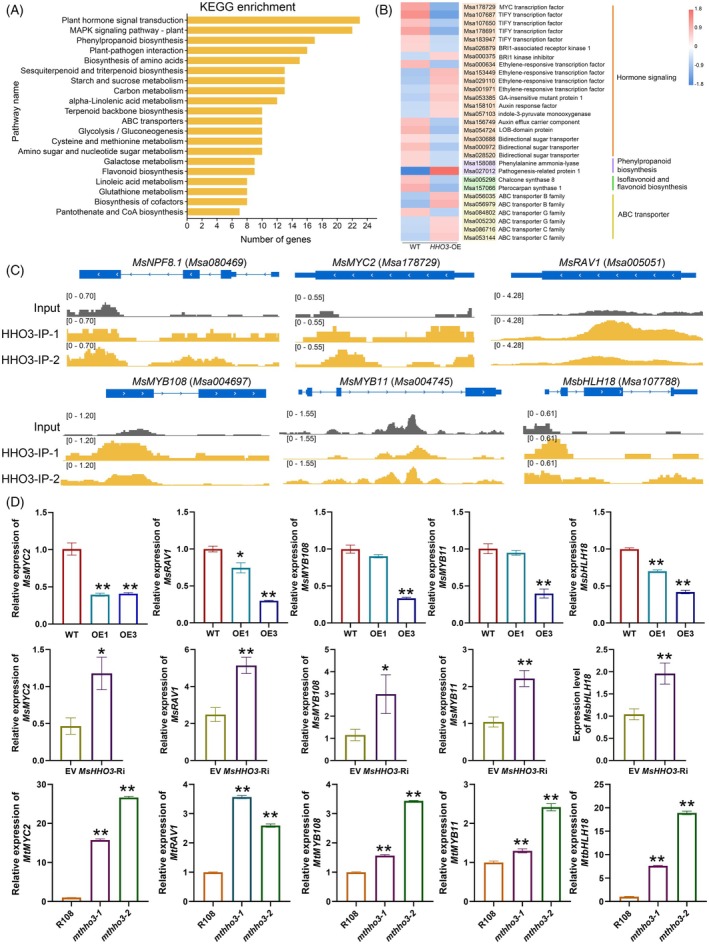
Identification of downstream genes of MsHHO3 via RNA‐Seq and ChIP‐Seq analysis. (A) Top 20 KEGG pathway enrichments of DEGs in alfalfa roots of WT and *MsHHO3‐*overexpressing plants. (B) Heatmap analysis of DEGs in SNF‐related pathways. (C) MsHHO3‐binding peaks in target genes (*P* < 0.01). (D) qRT‐PCR analysis of the direct targets of MsHHO3 in *MsHHO3*‐overexpressing lines, *MsHHO3*‐Ri and *mthho3* mutants, compared to the control. Two‐tailed Student's *t*‐test was used to identify significant differences (*P < 0.05, **P < 0.01).

To identify the downstream genes regulated by MsHHO3, RNA‐seq was performed using roots from WT and *MsHHO3*‐overexpressing plants inoculated with rhizobia for 10 days. Based on *P* < 0.05 and |log_2_(fold change)| ≥2, 2340 DEGs were identified, of which 1007 genes were upregulated and 1333 genes were downregulated. KEGG pathway enrichment analysis indicated that the DEGs were mainly enriched in plant hormone signal transduction and MAPK signaling pathways. Additionally, genes associated with plant–pathogen interactions, phenylpropanoid biosynthesis, ABC transporters, flavonoid biosynthesis, and glutathione metabolism also showed marked enrichment, all of which are correlated with SNF in leguminous plants (Figure 5A,B). Plant hormones are well‐known essential regulators of nodulation (Lin et al., 2020; Liu et al., 2018). The results showed that genes related to jasmonic acid (JA), brassinosteroid (BR), auxin, gibberellins (GA), and ethylene were differentially expressed, indicating that hormone signaling may play a crucial role in MsHHO3‐modulated SNF process (Figure 5B). Several genes previously reported to be important for nodulation were identified (Chen et al., 2025; Gao et al., 2021; Jarzyniak et al., 2021; Pawela et al., 2019; Roy et al., 2020; Sugiyama et al., 2017; Wang et al., 2019; Xiao et al., 2025). For instance, *YUC6*, *STR*, *PIN5*, *LBD21*, and *SWEETs*. qRT‐PCR results showed that the expression of *YUC6* and *STR* was significantly higher in *MsHHO3*‐overexpressing lines than in WT, but significantly lower in *MsHHO3*‐Ri and *mthho3* mutants compared to the control. The expression of *PIN5*, *LBD21*, and *SWEETs* is inhibited by MsHHO3. qRT‐PCR results further confirmed that these genes displayed significantly lower expression in *MsHHO3*‐overexpressing lines than in WT, while being significantly higher in *MsHHO3*‐Ri and *mthho3* mutants compared to the control (Figure S13).

To identify the direct targets of MsHHO3, ChIP‐seq was performed on roots from WT and *MsHHO3*‐overexpressing plants inoculated with rhizobia for 10 days. A total of 5107 MsHHO3 binding peaks were identified, encompassing 3222 genes, with 25.26% of the peaks located in promoters (Figure S14A). Detailed genome‐wide distribution analysis showed that MsHHO3 binding sites were highly enriched at the proximal locations of the transcription start sites (TSS) of genes (Figure S14B). Integrative analysis of ChIP‐seq and RNA‐seq data identified 85 genes as potential direct targets of MsHHO3, with 32 upregulated and 53 downregulated (Figure S14C). Among the 85 genes, several TF‐encoding genes were identified (Figure 5C), including the JA pathway master regulator, *MYC2* (*Msa178729*), *RAV1* (*Msa005051*), *bHLH18* (*Msa107788*), and two *MYBs* (*MYB108*:*Msa004697*, *MYB11*:*Msa004745*). In addition, one nitrogen transporter gene *Msa080469* (*NPF8.1*) was identified as being directly downregulated by MsHHO3, which is consistent with previous reports that NIGT1 family TFs modulate plant nitrogen utilization (Maeda et al., 2018; Wang, Wang, et al., 2020) (Figure 5C). qRT‐PCR analysis revealed that the expression of TF encoding genes was significantly altered in *MsHHO3*‐overexpressing lines, *MsHHO3*‐Ri, and *mthho3* mutants compared to the control (Figure 5D), suggesting that these TFs may play roles in MsHHO3‐mediated SNF process. Taken together, these findings suggest that MsHHO3 plays a critical role in SNF by directly or indirectly modulating multiple pathways.

### MsHHO3 directly modulates *MsMYC2*

**Figure 6:**
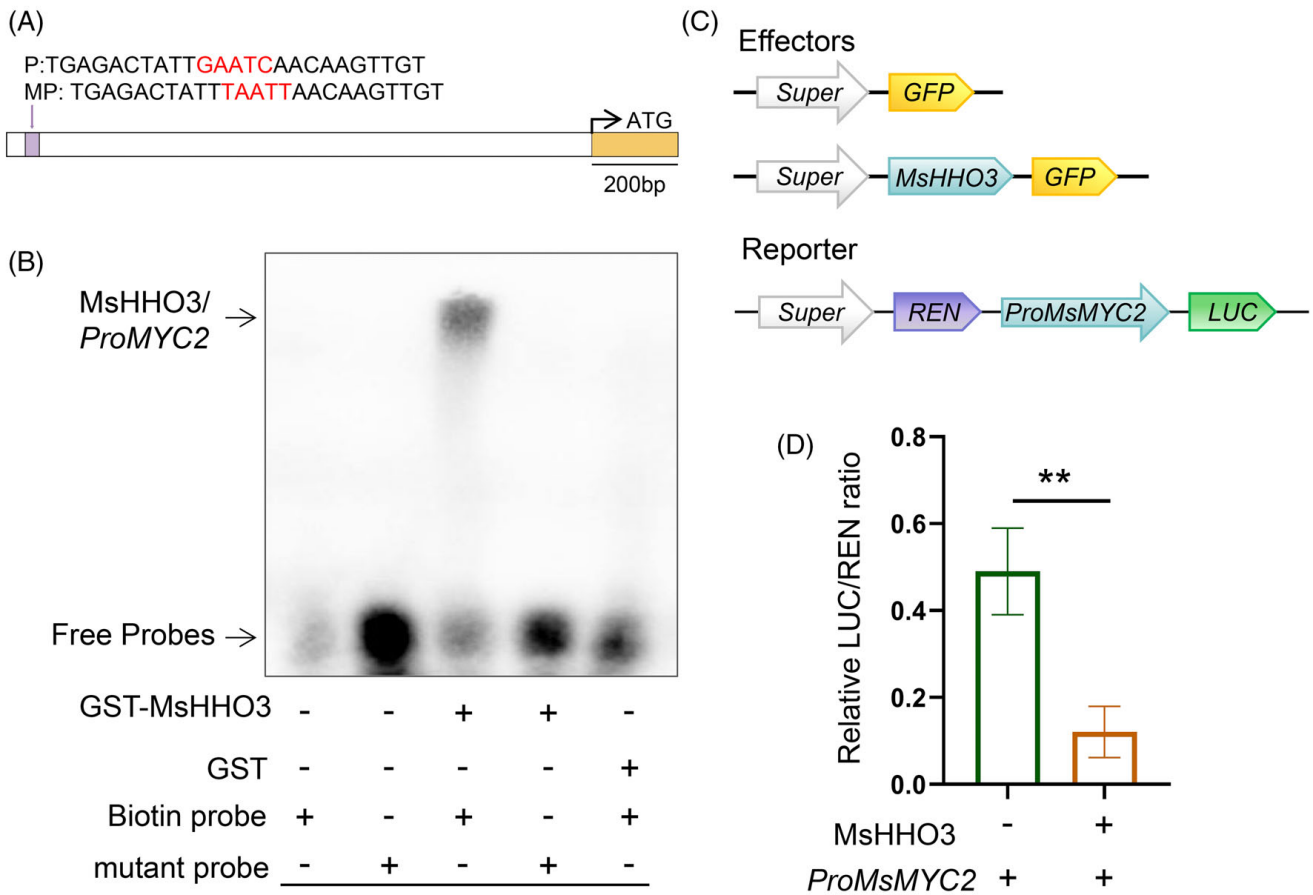
MsHHO3 directly modulates *MsMYC2*. (A) Schematic of *MsMYC2* promoter showing the *cis*‐element position. (B) EMSA assay of GST‐MsHHO3 protein and *MsMYC2* promoter *in vitro*. (C) Schematic diagram of the *MsHHO3* effector and *MsMYC2* reporter constructs used for dual‐luciferase assay. (D) Relative LUC activity from transient co‐expression of *Super:MsHHO3* and *ProMsMYC2:LUC* constructs in *Nicotiana benthamiana* leaves. Two‐tailed Student's *t*‐test was used to identify significant differences (**P < 0.01).

MYC2 is a key regulatory factor in the JA signaling pathway, capable of regulating plant growth by integrating different hormone signaling pathways (Kazan & Manners, 2013; Mu et al., 2025). Phylogenetic analysis showed that Msa178729 clusters with *M. truncatula* MtrunA17_Chr8g0346151 and MtrunA17_Chr8g0346161 which are annotated as MtMYC2 and MtMYC1, respectively. This subclade also contains Arabidopsis AtMYC2 and AtMYC3, as well as *M. truncatula* MtrunA17_Chr8g0366751, which is designated as MtMYC2 and has been reported to modulate nodule formation (Guo et al., 2024). Protein domain analysis revealed that Msa178729 contains a JID/JAZ interaction domain in the N‐terminus and a basic helixloop‐helix domain (Figure S15A). qRT‐PCR analysis showed that *Msa178729* is induced by both MeJA and mechanical damage, consistent with the characteristics of *AtMYC2* and *MtMYC2* (Guo et al., 2024; Zhang et al., 2020) (Figure S15B,C). Based on these results, Msa178729 was designated as MsMYC2. To further confirm whether the MsHHO3 protein binds to the promoter of *MsMYC2*, we first conducted an electrophoretic mobility shift assay (EMSA). It has been reported that, as repressors, NIGT1 family TFs modulate the expression of downstream genes by directly binding to *cis*‐elements containing the motifs GAATC and GAATATTC (Kiba et al., 2018; Maeda et al., 2018; Sawaki et al., 2013). Analysis of the 1338 bp promoter of *MsMYC2* revealed the presence of one GAATC motif (Figure 6A). EMSA results showed that the recombinant MsHHO3 protein binds to the region, with binding abolished when the motif was mutated (Figure 6B). In addition, integrated RNA‐Seq and ChIP‐Seq analysis identified 53 downstream repressive target genes of MsHHO3 (Figure S14C). Promoter sequences (2000 bp upstream of ATG start codon) were analyzed, revealing that 46 genes harbored GAATC binding motifs (Table S1). The results indicated that MsHHO3 represses gene expression primarily by binding to the *cis*‐elements containing the GAATC motif.

To further validate the regulation of MsHHO3 on *MsMYC2*, we conducted a dual‐luciferase reporter assay in *N. benthamiana* leaves. An effector construct expressing *MsHHO3* was co‐infiltrated with a reporter construct containing the *MsMYC2* promoter driving firefly luciferase (LUC), while Renilla luciferase (REN) under the 35S promoter served as an internal control (Figure 6C). The result showed that co‐expression of *MsHHO3* and *MsMYC2pro:LUC* significantly reduced LUC/REN activity, demonstrating that MsHHO3 suppresses *MsMYC2* promoter activity (Figure 6D).

## DISCUSSION

**Figure 7:**
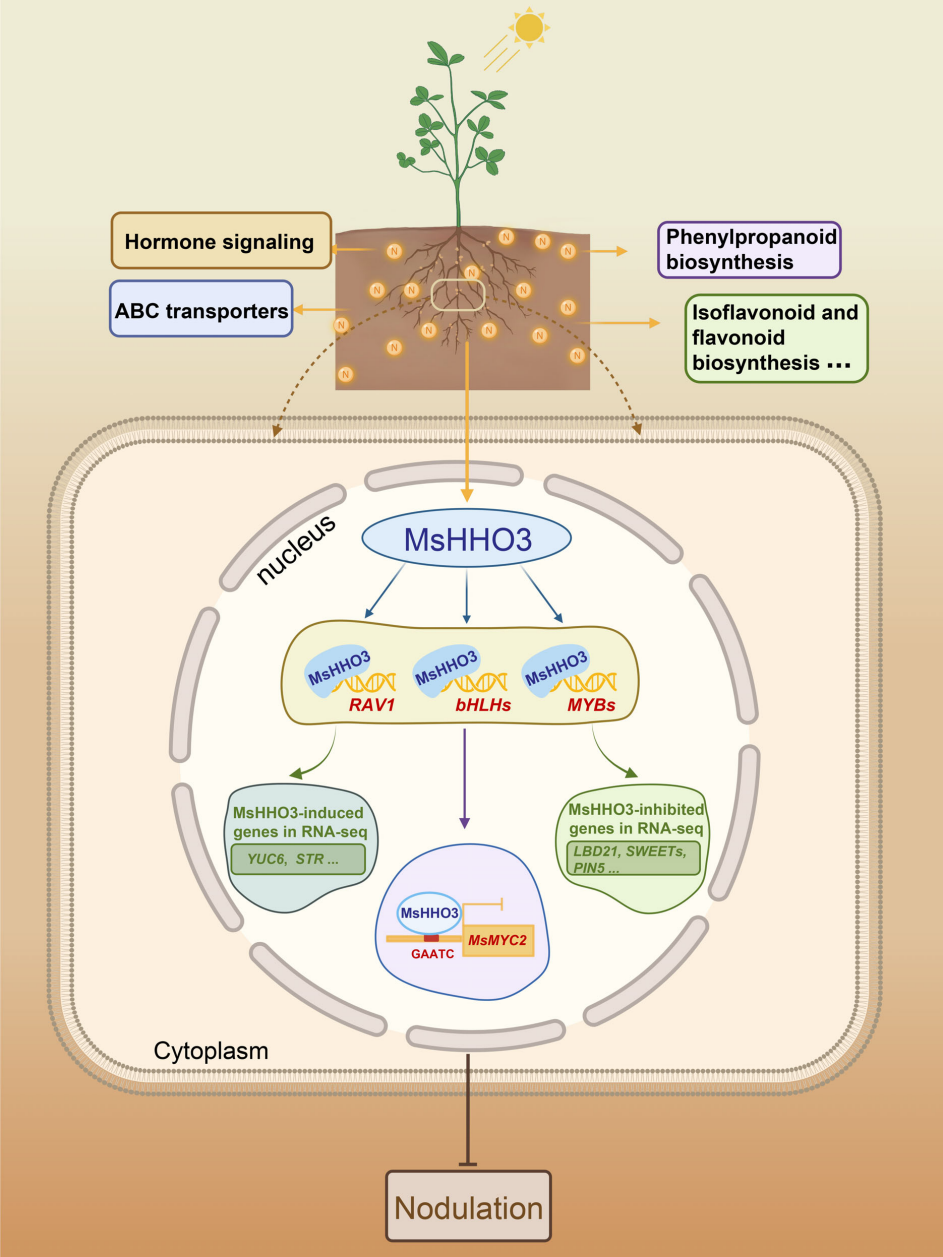
Regulatory module of MsHHO3‐mediated suppression of SNF in alfalfa under high N condition. Under high N, *MsHHO3* is induced in roots and nodules and it directly regulates other TF‐encoding genes, including *RAV1*, *MYC2*, *bHLH18*, *MYB11*, and *MYB108*. This direct regulation initiates a transcriptional cascade that modulates nodulation‐associated genes through indirect mechanisms.

Leguminous plants establish symbiotic relationships with rhizobia to convert atmospheric nitrogen into ammonium through SNF, which not only enhances soil nitrogen availability but also plays an important role in their yield and quality (Herridge et al., 2008). To adapt to fluctuating nitrate conditions, legumes have evolved sophisticated regulatory networks to fine‐tune SNF. In this study, we investigated the effects of elevated nitrate concentrations on alfalfa SNF, and MsHHO3 was identified to be a negative regulator in nitrate‐responsive nodulation and nitrogen fixation. *MsHHO3* expression was induced under high N conditions. High N treatment altered multiple pathways in nodules, including phenylpropanoid biosynthesis, plant hormone signal transduction, plant–pathogen interaction, glutathione metabolism, ABC transporters, etc. MsHHO3 was found to participate in these pathways, suggesting its important role in the SNF process of alfalfa. ChIP‐seq analysis demonstrated that MsHHO3 directly binds to the promoters of several TF‐encoding genes, including *MsRAV1*, *MsbHLHs*, and *Ms* *MYBs*. We propose that MsHHO3 functions through an intermediate TF‐mediated transcriptional cascade, thereby coordinating the expression of key nodulation‐related genes such as *YUC6*, *STR, PIN5, LBD21*, and *SWEETs* to precisely regulate SNF. Notably, we identified *MYC2*, a master regulator of JA signaling and nodulation, as a direct target. Further validation through EMSA and dual‐luciferase assays confirmed that MsHHO3 binds to the *MsMYC2* promoter, establishing a novel MsHHO3‐*MsMYC2* regulatory module (Figure 7).

bZIP11 and HHO3 are key regulators in N utilization (Ueda et al., 2020), the bZIP11‐HHO3 regulatory axis, known in rice, appears conserved in alfalfa nodule. In our study, MsbZIP11 emerged as a highly connected hub TF interacting with MsHHO3. In legumes, bZIP11 modulates both nodule formation and senescence (D'haeseleer et al., 2010; Van Dingenen et al., 2023). The NIGT1 subfamily TFs are widely involved in plant nitrogen signaling pathways and are critical regulators of nitrogen starvation responses, playing an important role in the regulation of nitrogen uptake and utilization. For example, in Arabidopsis, *NIGT1.1* (*HHO3*) is induced by nitrate and regulates the high‐affinity NITRATE TRANSPORTER2.1 (*NRT2.1*) (Kiba et al., 2018). As a NIGT1 family member, *MsHHO3* showed nitrate‐dependent induction and significant differential expression between the high‐nodulating TS and low‐nodulating LA alfalfa cultivars. Taken together, we hypothesize that MsHHO3 regulates nitrate‐responsive SNF.

MsHHO3 is localized in the nucleus and upregulated by high N. The *MsHHO3*‐overexpressing lines exhibited a significant reduction in nodule number, nodule fresh weight, and nitrogenase activity. Conversely, *MsHHO3*‐Ri and *mthho3* knockout mutants both displayed increased nodule number, nodule fresh weight, and enhanced nitrogenase activity. It has been reported that soybean has four NIGTs, namely GmNIGT1a, GmNIGT1b, GmNIGT1c, and GmNIGT1d (Zhou et al., 2024). GmNIGT1a and GmNIGT1b are localized in both the nucleus and cytoplasm and function as transcriptional repressors in soybean. Overexpression of *GmNIGT1a* resulted in decreased nodule number and weight. However, no significant phenotypic difference was observed between *nigt1ab* double mutant and wild‐type. Through yeast one‐hybrid and EMSA assays, GmNIGT1a and GmNIGT1b were proposed to modulate the expression of *GmNRAMP2a* and *GmNRAMP2b*, which are involved in iron (Fe) transport (Zhou et al., 2024). Our data indicate that HHO3 in Medicago acts as both an activator and a repressor. It primarily participates in hormone signal transduction and modulates SNF under high N conditions. The observed differences among NIGT1 family members across various species may arise from the fundamental distinctions between indeterminate and determinate nodule types. These nodule types exhibit distinct developmental patterns and are governed by different regulatory mechanisms underlying SNF (Guo et al., 2024; Kohlen et al., 2018; Lin et al., 2020; Tu et al., 2024).

Integrated analysis of RNA‐Seq and ChIP‐Seq data identified 85 candidate genes directly regulated by MsHHO3. Notably, a subset of these genes encodes TFs, all of which are potential regulators involved in hormone signaling pathways (Cui et al., 2018; Feng et al., 2014; Mandal et al., 2023; Mandaokar & Browse, 2009; Ren et al., 2018; Xu et al., 2019, 2022). In *M. truncatula*, MtMYC2 functions as a key positive regulator in SNF. The *mtmyc2* mutant displays reduced nodule weight and nitrogenase activity (Guo et al., 2024). In this study, *MsMYC2* exhibited expression patterns consistent with those of *MtMYC2* following MeJA treatment and mechanical wounding. Additionally, *MYC2* expression was significantly downregulated in *MsHHO3*‐overexpressing plants and upregulated in *MsHHO3*‐Ri and *mthho3* mutants. Co‐expression of MsHHO3 with *MsMYC2pro:LUC* significantly suppressed LUC/REN activity. Previous studies demonstrated that NIGT1 family proteins bind to GAATC and GAATATTC elements to repress the expression of downstream genes (Kiba et al., 2018; Maeda et al., 2018; Sawaki et al., 2013). We identified one GAATC motif in the promoter of *MsMYC2*. EMSA analysis showed that MsHHO3 can directly bind to GAATC element. These results demonstrate that MsHHO3 may at least partially suppress SNF by directly regulating *MsMYC2* in alfalfa. The HHO3‐*MYC2* module provides novel insights into nitrate‐mediated nodulation in alfalfa.

Furthermore, RNA‐Seq analysis of the *MsHHO3*‐overexpressing line identified a series of differentially expressed genes, some of which are potentially involved in alfalfa nodulation, such as *YUC6*, *STR*, *PIN5*, *LBD21*, *SAUR50*, and *SWEETs* (Figure S13), whose family members have been reported to participate in SNF (Chen et al., 2025; Gao et al., 2021; Jarzyniak et al., 2021; Pawela et al., 2019; Roy et al., 2020; Sugiyama et al., 2017; Wang et al., 2019; Xiao et al., 2025). Although MsHHO3 does not directly regulate these genes, sequence analysis revealed the presence of *cis*‐elements recognized by other TFs, such as G‐box (CACGTG) recognized by MYC2 (Guo et al., 2024), CAACA recognized by RAV1 (Feng et al., 2014). This suggests that these genes are likely direct targets of the intermediate TFs. Thus, we propose that the SNF suppression mediated by MsHHO3 not only relies on its direct regulation of downstream genes, but also involves a broader transcriptional cascade network driven by intermediate TFs.

## MATERIALS AND METHODS

### Plant materials and growth conditions

The alfalfa (*M. sativa* L.) cv. Zhongmu No. 4 and *M. truncatula* R108 were used as wild‐type materials for nodulation assays and genetic transformation. For nodulation analysis of wild‐type alfalfa, vegetatively propagated cuttings were grown in vermiculite for 2 weeks, followed by selection of uniformly growing seedlings that were transplanted into a perlite: vermiculite (5:2, v/v) mixture presoaked in N‐deficient Fåhraeus nutrient solution. After 1 week of acclimation in this medium, plants were inoculated with *Sinorhizobium meliloti* strain *Sm1021* (OD_600_ = 0.05). Two weeks postinoculation, to analyze the effects of different nitrogen levels on nodulation, the plants were treated with nutrient solutions supplemented with 0, 2, or 10 mM KNO_3_, respectively. For the N‐deficient nodulation assay involving alfalfa overexpression lines, RNAi lines, and *M. truncatula* mutants, plants were subjected to identical cultivation and inoculation conditions as those applied to wild‐type plants above, with nodule phenotypes characterized at 14 dpi. For alfalfa RNAi hairy root lines under high N conditions, 3‐week‐old plants were transferred into a perlite: vermiculite (5:2, v/v) mixture irrigated with nutrient solution containing 8 mM KNO_3_. After 1 week of growth, plants were inoculated with *S. meliloti* strain *Sm1021* (OD_600_ = 0.05) and cultivated in the greenhouse for a further 3 weeks to analyze. For nodulation analysis of *M. truncatula* mutants under high N conditions, seeds were first surface‐sterilized with 10% sodium hypochlorite solution for 10 min, washed five times with distilled water, and germinated on moist filter paper. The subsequent treatment conditions were subjected to the same treatment conditions as the alfalfa RNAi lines described above.

### Plasmid construction and plant transformation

The full‐length CDS without a stop codon of *MsHHO3* was amplified using primers *MsHHO3*‐OE‐F and *MsHHO3*‐OE‐R and cloned into the *Super1300: GFP* expression vector. Stable transgenic alfalfa lines were generated via *Agrobacterium tumefaciens* strain EHA105‐mediated transformation, as previously described (Fu et al., 2015). For the assembly of RNAi constructs, a 200‐bp fragment specific to the *MsHHO3* CDS was amplified and recombined into the pK7GWIWG2D(II) vector through Gateway™ LR clonase (Invitrogen) reactions. The *MsHHO3*‐Ri hair roots were generated via *A. rhizogenes* strain 1193‐mediated transformation, as previously described (Boisson‐Dernier et al., 2001).

For the *mthho3* mutants, a 20‐bp sgRNA (M: TCGTGACTATATCCTAGCATTGG) target in the first exon of *MtHHO3* was designed using the CRISPR‐Cas9 system and cloned into the *pHEE401* vector (Wang et al., 2015). Homozygous mutant plants were generated via *A. tumefaciens* strain EHA105‐mediated transformation in *M. truncatula* wild‐type R108, and seeds were harvested for further assays.

### Expression pattern analysis of *MsHHO3*

The vegetatively propagated cuttings of alfalfa cv. Zhongmu No. 4 were grown under normal conditions at 24/22°C, 16/8 h light/dark photoperiod, and 60% relative humidity for 1 month, and the fresh samples of roots, nodules, stems, and leaves were harvested for analysis of gene tissue expression.

Stem cuttings of alfalfa cv. Zhongmu No. 4 were rooted in vermiculite for 2 weeks, then transferred to 1/2 Hoagland nutrient solution. After a 3‐day acclimation period, the plants were transferred to Fåhraeus nutrient solution supplemented with 0 mM or 10 mM KNO_3_, respectively, and cultured for 1 week before inoculation with *S. meliloti* strain *Sm1021* (OD_600_ = 0.05). Whole roots (including nodules) were sampled at 5, 10, 21, 30, and 45 dpi for gene expression analysis.

The 2000 bp promoter of *MtHHO3* was amplified and cloned into the *pCAMBIA1381* vector to drive expression of the β‐glucuronidase GUS reporter gene. The recombinant construct was introduced into *A. tumefaciens* strain EHA105 and stable transgenic lines of *M. truncatula* were generated (Fu et al., 2015). For GUS staining, *ProMtHHO3:GUS* transgenic plants were grown in a perlite:vermiculite (5:2, v/v) mixture soaked in nutrient solution containing 0 or 10 mM KNO_3_. After 7 days of treatment, plants were inoculated with *S. meliloti* strain *Sm1021* (OD_600_ = 0.05). Roots with nodules were harvested 21 dpi and infiltrated with GUS staining buffer containing 79.9 μM phosphate buffer, 0.5 mM potassium ferrocyanide, 0.5 mM potassium ferricyanide, 2 μl Triton X‐100 and 1 mg X‐Gluc, followed by overnight incubation at 37°C in darkness. Samples were decolorized with 75% ethanol, and the images were captured using a Leica microscope (M205 FA). The decolorized nodules were embedded in agar and sectioned into 60‐μm‐thick slices using a vibratome, and the sections were scanned for microscopic analysis.

### Subcellular localization of MsHHO3

The full‐length CDS of *MsHHO3* without a stop codon was cloned into the *Super1300: GFP* vector, with the empty vector used as a control. The verified construct, together with the nuclear marker mRFP‐AHL2, was transiently co‐expressed in *N. benthamiana* leaves via *A. tumefaciens* strain GV3101‐mediated infiltration. Fluorescence was detected 48 h after infiltration.

For subcellular localization in alfalfa root and nodule, vegetatively propagated cuttings of *MsHHO3*‐overexpression lines were rooted in vermiculite for 2 weeks, transferred to 1/2 Hoagland nutrient solution for 3 days, and then cultured in Fåhraeus nutrient solution (0 mM KNO₃) for 7 days before inoculation with *S. meliloti* strain *Sm1021* (OD_600_ = 0.05). At 14 dpi, plants were transferred to Fåhraeus solution containing either 0 mM or 10 mM KNO₃ for treatment. Then root segments were mounted for GFP signal observation. The nodules were embedded in agar, sectioned into 30 μm slices with a vibratome, and stained with 1 μg ml^−1^ DAPI for 10 min in the dark at room temperature. After rinsing with PBS, fluorescent signals were observed. All fluorescence imaging was detected using a Nikon confocal laser scanning microscope (Nikon C2‐ER).

### Nitrogenase activity measurement

Nitrogenase activity was quantified via the acetylene reduction assay (ARA), following the methods described previously (Hardy et al., 1968; Luo et al., 2023). The plant roots were sampled, and three plants per group were placed into 100 ml glass vials sealed with rubber stoppers. A 10 ml of air was withdrawn from each vial, and an equal volume of acetylene gas was injected. The vials were then incubated at 28°C for 2 h. After incubation, 2 ml of air was removed from the serum vials with 1 ml ddH₂O, and an equal volume of gas from the incubated vials was injected. The serum vials were immediately sealed, inverted, submerged in water for liquid sealing, and reserved for analysis. Then, 100 μl of ethylene gas was analyzed via the gas chromatograph which served as an indicator of nitrogenase activity. The nodules were detached from the roots and their fresh weights were measured. Nitrogenase activity was expressed as nanomoles of ethylene produced per gram of nodule fresh weight per hour (nmol C_2_H_4_ g^−1^ h^−1^).

### Phylogenetic analysis

For the phylogenetic analysis of MsHHO3, protein sequences from the NIGT1/HRS1/HHO subfamily of *A. thaliana*, *M. truncatula*, and *M. sativa* were aligned using the ClustalW algorithm in the MEGA (Molecular Evolutionary Genetic Analysis) 11.0 software. The neighbor‐joining evolutionary tree was constructed with 1000 bootstrap replicates and gap and missing data were treated via complete deletion.

### RNA extraction and quantitative real‐time PCR (qRT‐PCR)

Total RNA was extracted from fresh plant tissues using the Eastep Super Total RNA Extraction KIT (Promega, USA) and quantified with a NanoPhotometer NP80 (Implen, Germany). Frist‐strand cDNA was synthesized from 1 μg of total RNA using the HiScript III All‐in‐one RT SuperMix Perfect (Vazyme, China). Amplifications were performed using CFX‐96 real time system (Bio‐Rad) with SYBR qPCR Master Mix (Vazyme, China). Relative gene expression was quantified via the 2^−∆∆CT^ method. The primer sequences were listed in Table S2.

### RNA sequencing

The uniform seedlings of WT and *MsHHO3*‐overexpressing lines treated with N‐deficient Fåhraeus nutrient solution for 1 week were inoculated with *S. meliloti* strain *Sm1021* (OD_600_ = 0.05) and grown in greenhouse for 10 days, and the roots were harvested for total RNA extraction. cDNA libraries were subsequently prepared using qualified RNA samples. cDNA libraries were pooled and sequenced on the Illumina NovaSeq 6000 system. Clean reads were obtained by removing adapter‐containing reads, poly‐N sequences, and low‐quality reads from raw reads. Then, clean reads were aligned to the alfalfa reference genome (Zhongmu No. 4) using HISAT2 (v2.0.5). Expression quantification was conducted using feature Counts (v1.5.0‐p3), with expression levels normalized by the fragments per kilobase of transcript per million mapped fragments (FPKM) method. Comparative analysis of differential expression between WT and *MsHHO3*‐overexpressing lines was performed through the DESeq2 R package (v1.20.0). Adjusted *P* values (*P*) were calculated via the Benjamini–Hochberg method to control the false discovery rate (FDR), with DEGs identified by using thresholds of *P* < 0.05 and |log_2_(fold change)| ≥1.

### WGCNA analysis and Transcription factors regulatory network construction

Co‐expression network was constructed using the standard gene set and the WGCNA package in R (Version 3.5.0), with the threshold set at 0.8. The hub genes were identified based on module membership value (KME) ≥0.85, and 82 TF‐encoding genes were included. A protein–protein interaction (PPI) network for the 82 TFs was constructed using the STRING database (version 12.0). The search was performed against the reference proteome of *M. sativa* (Zhongmu No. 4; STRING taxon identifier STRG0A86XBD). The “full STRING network” setting was used, with edges indicating both functional and physical associations. The resulting PPI network was clustered within STRING using the k‐means method. Network data were downloaded in TSV format and imported into Cytoscape (version 3.8.2) for visualization and topological analysis. One cluster containing four TFs, which lacked connections to the main network, was removed. The connectivity of each node was quantified using the “Degree” metric, defined within Cytoscape as the number of edges linked to that node.

### ChIP‐seq assay

The uniform seedlings of WT and *MsHHO3*‐overexpressing lines treated with N‐deficient Fåhraeus nutrient solution for 1 week were inoculated with *S. meliloti* strain *Sm1021* (OD_600_ = 0.05) and grown in greenhouse for 10 days; the roots were harvested for ChIP‐seq assay. Purified DNA was used for ChIP‐seq library preparation sequenced in paired‐end mode on the Illumina platform (Illumina, CA, USA). The filtered clean reads were aligned to the alfalfa reference genome (Zhongmu No. 4) using BWA‐MEM (v0.7.12). IP‐enriched regions were identified with MACS2 (v2.1.0) using a q‐value threshold of 0.05 for peak calling against background signals, and visualization in Integrative Genomics Viewer (IGV).

### Dual‐luciferase reporter assays

The 1338 bp promoter sequence of *MsMYC2* was amplified from *M. sativa* Zhongmu No. 4 genomic DNA and cloned into the *pGreenII0800‐LUC* vector to generate the construct *ProMsMYC2:LUC*. The effector construct was *Super:MsHHO3‐GFP*, and the empty vector *Super:GFP* was used as a control. *A. tumefaciens* strain (GV3101 + PSOUP‐P19) harboring reporter and effector were infiltrated into the *N. benthamiana* leaves. After infiltration for 48 h, the LUC and REN activities were analyzed using Dual Luciferase Reporter Assay System (Promega, USA). The LUC activity was quantified based on the LUC/REN ratios.

### EMSA assays

The full‐length CDS of *MsHHO3* was amplified and cloned into the *pGEX‐4T‐2* vector. The fusion protein GST‐MsHHO3 was induced in *Escherichia coli* strain BL21 by adding 0.2 mM IPTG at 18°C for 10 h, and purified using Glutathione Sepharose 4B resin (GE Healthcare). The empty vector *pGEX‐4T‐2* was also introduced into *E. coli* to generate GST protein as control. EMSA was performed using the LightShift Chemiluminescent EMSA Kit (ThermoFisher Scientific) according to the manufacturer's instruction. The reactions were loaded onto a 6% native polyacrylamide gel in 0.5 × TBE buffer for electrophoresis. The probes used are listed in the Table S2.

### Statistical analysis

Statistical significance was determined by Student's *t*‐test and one‐way analysis of variance (ANOVA) at *P* < 0.05. Data were presented as mean ± SE. The figures were created using GraphPad Prism version 9.5 and BioRender software.

## AUTHOR CONTRIBUTIONS

Xue Wang, Qingchuan Yang, and Pengbo Liang designed the project. Yajing Wu, Qian Liu, Siqi Wang, Yuxuan Ding, and Xue Wang performed the experiments. Yajing Wu, Xue Wang, Qian Liu, and Pengbo Liang analyzed the data. Yajing Wu and Xue Wang wrote the manuscript. Xue Wang, Qingchuan Yang, and Pengbo Liang revised the manuscript. Fei He and Junmei Kang provided the alfalfa varieties.

## CONFLICT OF INTEREST

The authors have not declared a conflict of interest. No human or animal participants were conducted in this study.

## Supporting information


**Figure S1.** Top 20 KEGG pathway enrichments of DEGs in alfalfa nodules under varied nitrate treatments.
**Figure S2.** GO enrichment analysis of the turquoise, brown, and blue modules.
**Figure S3.** Nodules of TS and LA varieties with *pNifH:GUS* staining under N‐deficient conditions.
**Figure S4.** Nodule phenotypes of TS and LA varieties under high N conditions.
**Figure S5.** Expression analysis of red‐highlighted nonsignificantly different genes between TS and LA alfalfa varieties.
**Figure S6.** Characterization of MsHHO3.
**Figure S7.** Expression and nodule phenotype analysis of *MsHHO3*‐overexpressing lines.
**Figure S8.** Nodules of EV and *MsHHO3*‐Ri lines with *pNifH:GUS* staining under high N conditions.
**Figure S9.** Phenotypic comparison of EV and *MsHHO3*‐Ri under N‐deficient conditions.
**Figure S10.** Construction of the *mthho3* mutant.
**Figure S11.** Nodules of wild‐type (R108) and *mthho3* mutants with *pNifH:GUS* staining under high N conditions.
**Figure S12.** Nodule phenotypes of wild‐type (R108) and *mthho3* mutants under N‐deficient conditions.
**Figure S13.** qRT‐PCR analysis of nodulation gene expression in *MsHHO3* transgenic and mutant plants.
**Figure S14.** Integrated analysis of RNA‐Seq and ChIP‐Seq.
**Figure S15.** Characteristics of MsMYC2.

**Table S1.** Number of MsHHO3‐bound cis‐motifs in promoters of suppressed target genes.

**Table S2.** Primer sequences used in the present study.

## Data Availability

All data are presented in the manuscript and figures, with additional supporting information provided in the Supplementary Materials. Transcriptome data have been uploaded to the NCBI Sequence Read Archive (SRA) under the BioProject accession number PRJNA1387176.
