# Supplementary material for: Identification of symbiotic nitrogen fixation‐modulating factors in alfalfa and mechanism elucidation of MsHHO3
Source: Plant J. 2026 Mar 29;126(1):e70831. doi: 10.1111/tpj.70831 (PMC13033166; doi:10.1111/tpj.70831)
Supplement: Supplementary file 1 — Figure S1. Top 20 KEGG pathway enrichments of DEGs in alfalfa nodules under varied nitrate treatments. Figure S2. GO enrichment analysis of the turquoise, brown, and blue modules. Figure S3. Nodules of TS and LA varieties with pNifH:GUS staining under N‐deficient conditions. Figure S4. Nodule phenotypes of TS and LA varieties under high N conditions. Figure S5. Expression analysis of red‐highlighted nonsignificantly different genes between TS and LA alfalfa varieties. Figure S6. Characterization of MsHHO3. Figure S7. Expression and nodule phenotype analysis of MsHHO3‐overexpressing lines. Figure S8. Nodules of EV and MsHHO3‐Ri lines with pNifH:GUS staining under high N conditions. Figure S9. Phenotypic comparison of EV and MsHHO3‐Ri under N‐deficient conditions. Figure S10. Construction of the mthho3 mutant. Figure S11. Nodules of wild‐type (R108) and mthho3 mutants with pNifH:GUS staining under high N conditions. Figure S12. Nodule phenotypes of wild‐type (R108) and mthho3 mutants under N‐deficient conditions. Figure S13. qRT‐PCR analysis of nodulation gene expression in MsHHO3 transgenic and mutant plants. Figure S14. Integrated analysis of RNA‐Seq and ChIP‐Seq. Figure S15. Characteristics of MsMYC2. [file TPJ-126-0-s002.doc]

**Supplementary Material**

**Identification of** **symbiotic nitrogen fixation-modulating factors in alfalfa and mechanism elucidation of MsHHO3**

Yajing Wu^1^, Qian Liu^2,3^, Fei He^1^, Siqi Wang^1^, Yuxuan Ding^1^, Junmei Kang^1^, Pengbo Liang^2,3,^*, Qingchuan Yang^1,^*, Xue Wang^1,^*

^1^Institute of Animal Science, the Chinese Academy of Agricultural Sciences; Beijing 100193, China.

^2^ State Key Laboratory of Plant Environmental Resilience, Frontiers Science Center for Molecular Design Breeding (MOE), College of Biological Sciences, China Agricultural University; Beijing 100193, China.

^3^ MOA Key Laboratory of Soil Microbiology, and Rhizobium Research Center, China Agricultural University; Beijing 100193, China.

*Corresponding authors: pbliang@cau.edu.cn, qchyang66@163.com, [wangxue01@caas.cn](mailto:wangxue01@caas.cn).

**Figure S1.**

**
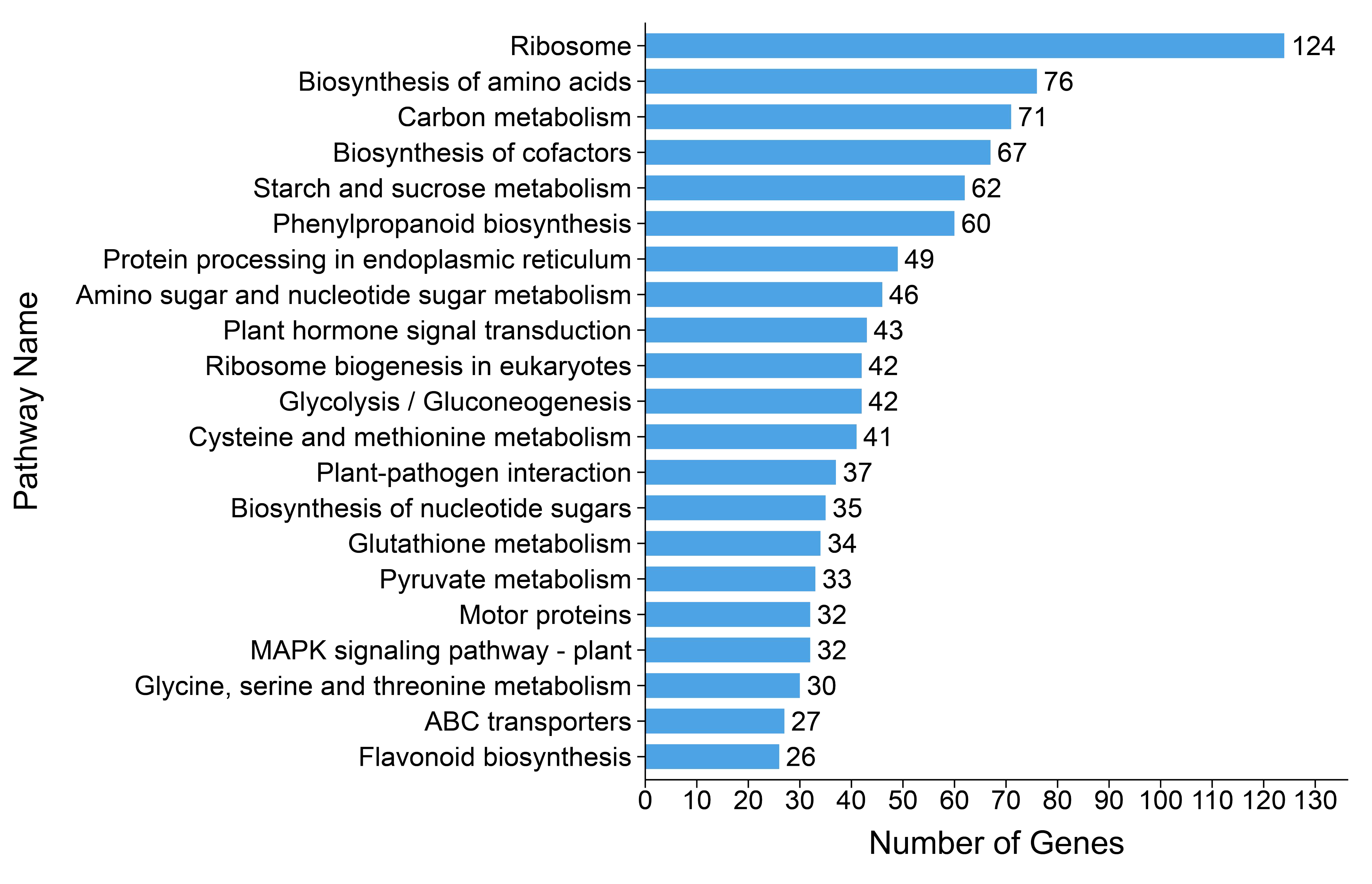
**

**Figure S1.** Top 20 KEGG pathway enrichments of DEGs in alfalfa nodules under varied nitrate treatments.

**Figure S2.**

**
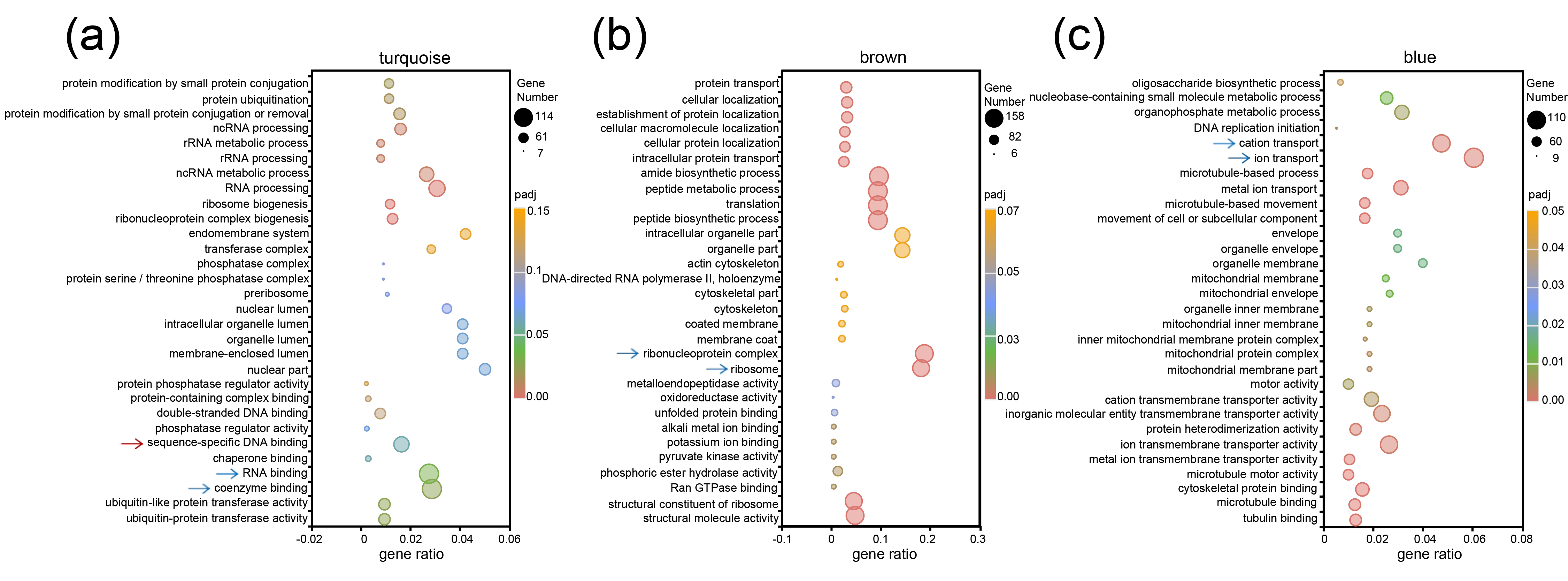
**

**Figure S2.** GO enrichment analysis of the turquoise, brown, and blue modules, padj < 0.05.

**Figure S3.**

**
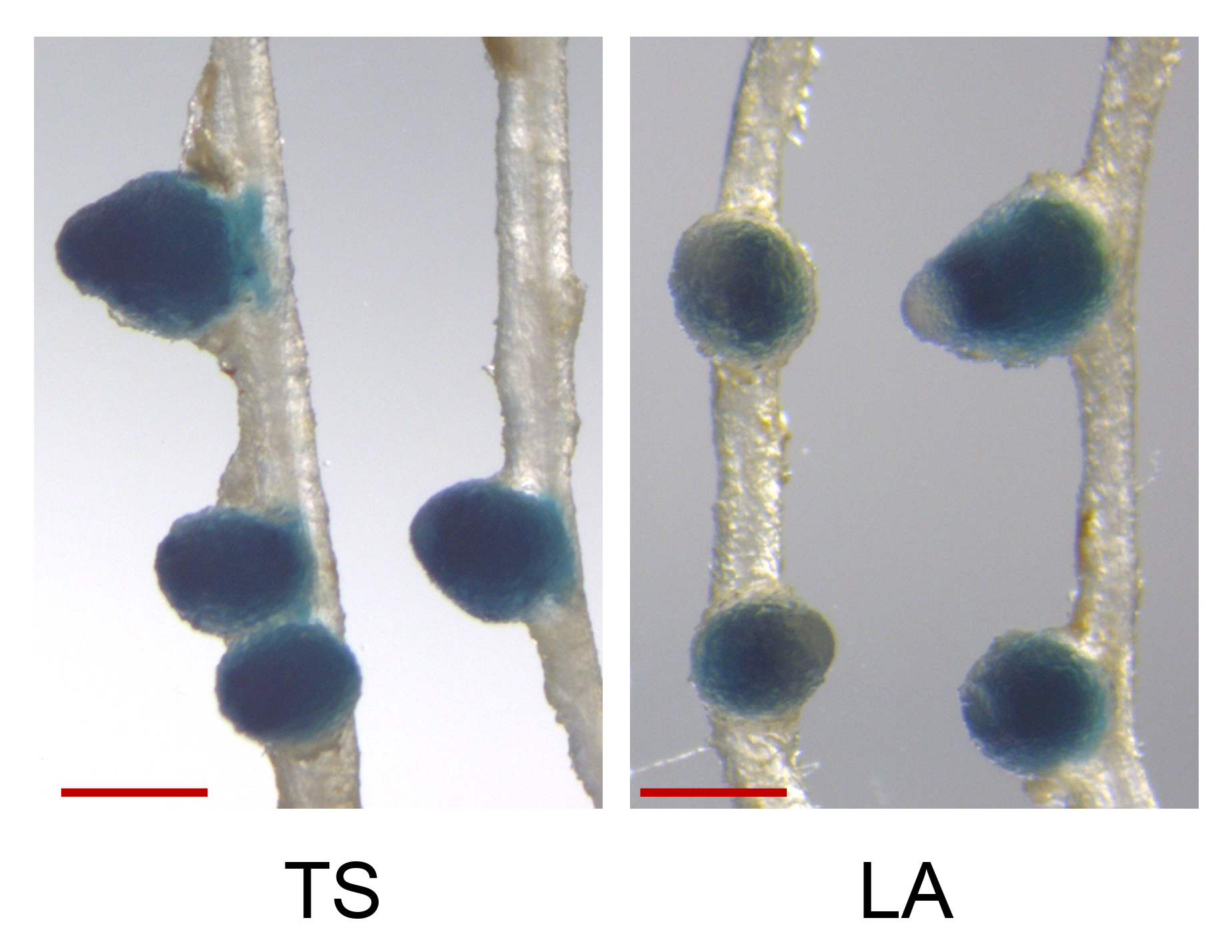
**

**Figure S3.** Nodules of TS and LA varieties with *pNifH:GUS* staining under N-deficient conditions. Scale bar=1 mm.

**Figure S4.**

**
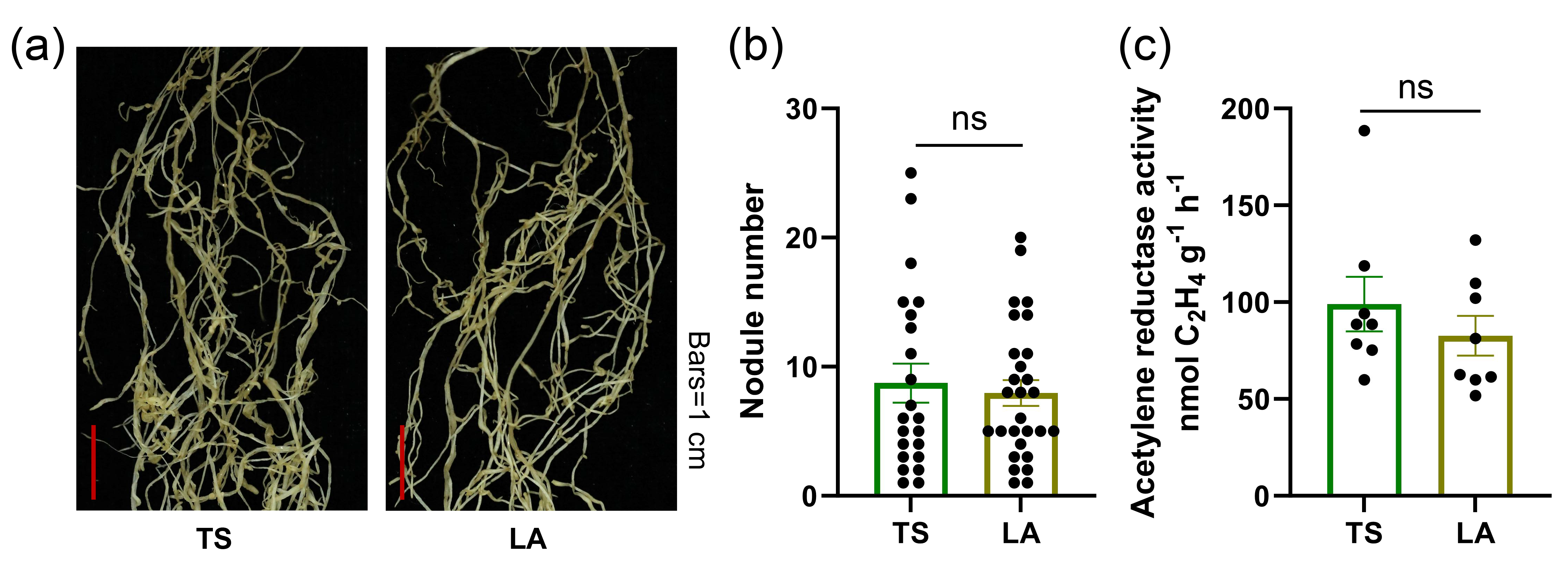
**

**Figure S4.** Phenotypic comparison of TS and LA varieties under high N conditions. (a) Nodule phenotypes of TS and LA. Scale bar=1 cm. (b) Nodule number. (c) Nitrogenase activity. Two-tailed student's t-test was used to identify significant differences.

**Figure S5.**

**
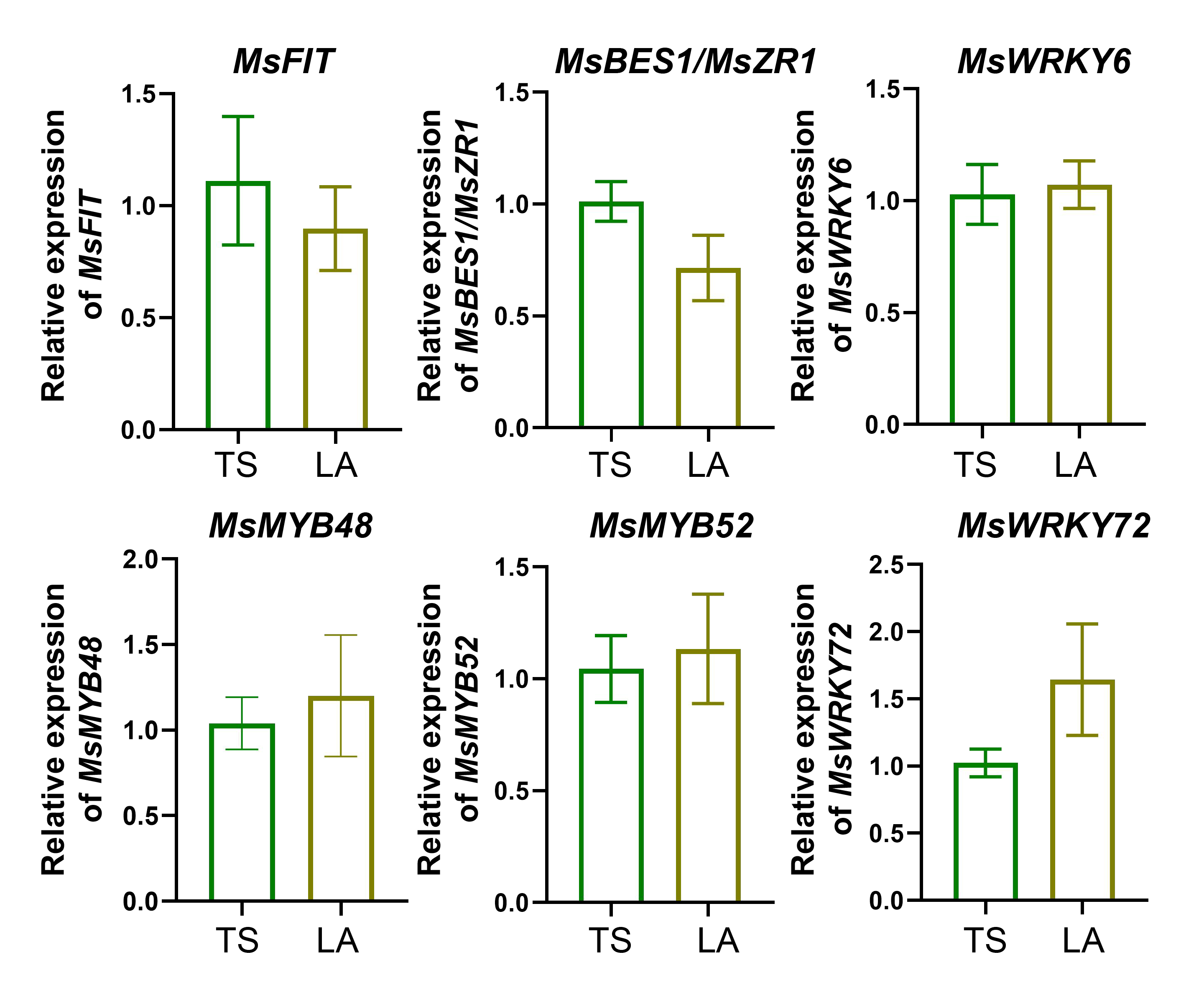
**

**Figure S5.** Expression analysis of red-highlighted non-significantly different genes between TS and LA alfalfa varieties.

**Figure S6.**


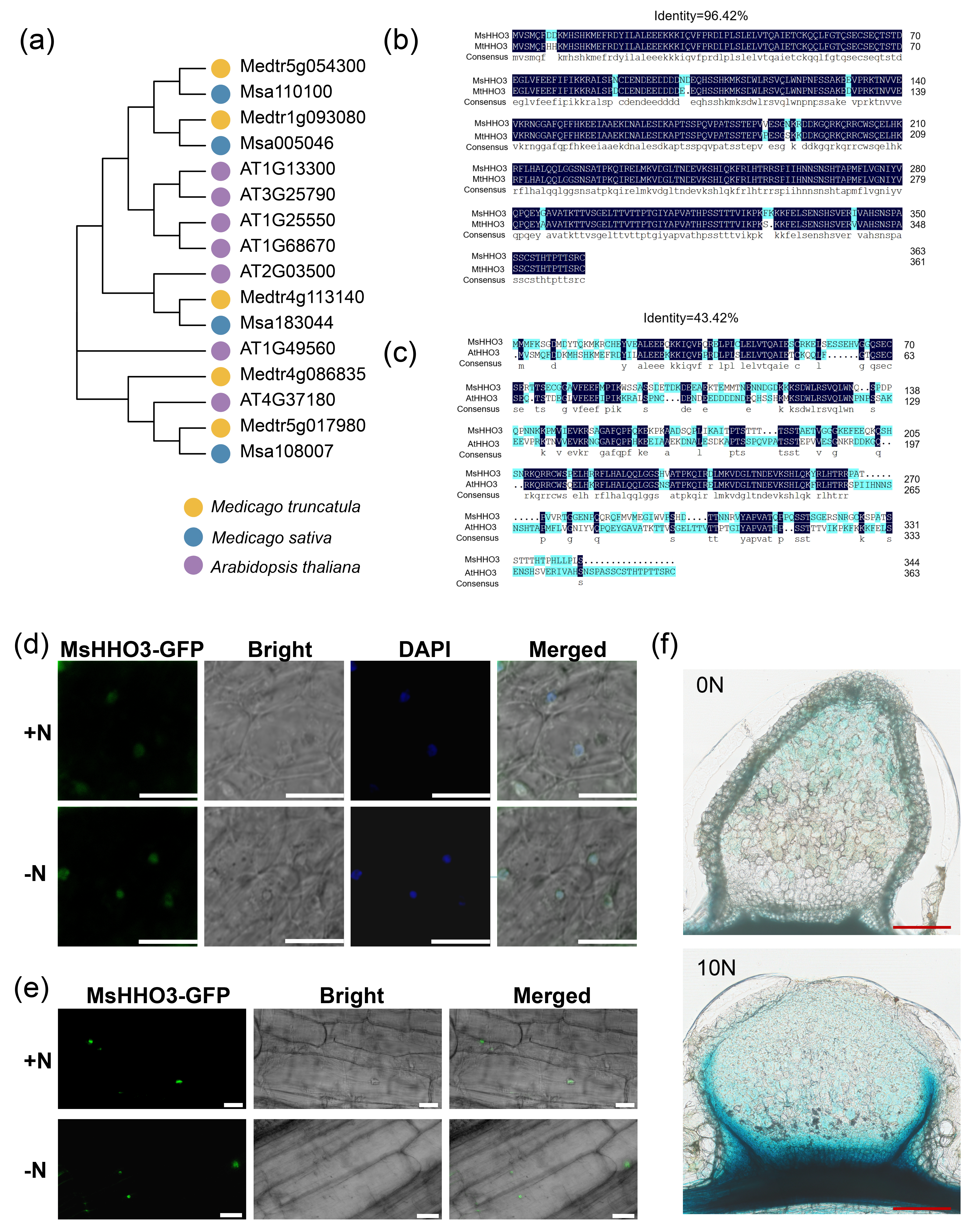


**Figure S6.** Characterization of MsHHO3.

(a) Phylogenetic tree of NIGT1/HRS1/HHO family proteins in *Medicago sativa*, *Medicago truncatula* and *Arabidopsis thaliana.* Species are distinguished by blue, yellow, and purple circles, respectively. (b) Amino acid sequence alignment between MsHHO3 and MtHHO3. (c) Amino acid sequence alignment between MsHHO3 and AtHHO3. The percentage of identity reflects differences in homology. Conserved amino acid residues are highlighted, and the consensus sequence is displayed below the alignment. (d) Subcellular localization of MsHHO3 in alfalfa nodule cells. Scale bar=20μm. (e) Subcellular localization of MsHHO3 in alfalfa root cells. Scale bar=20μm. (f) Longitudinal sections of nodules from stable *ProMtHHO3-GUS* transgenic lines under 0N and 10N conditions. Scale bar=200μm.

**Figure S7.**


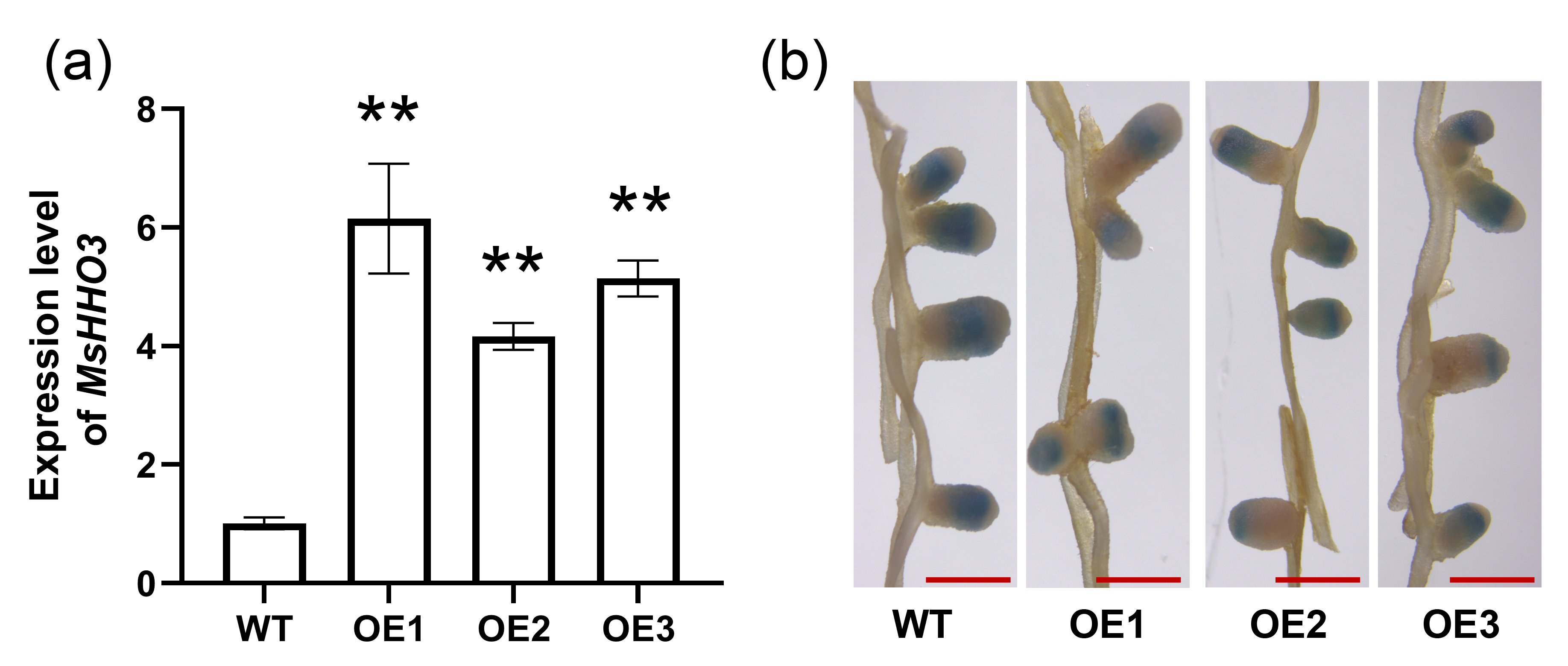


**F****igure S7.** Expression and nodule phenotype analysis of MsHHO3-overexpressing lines.

1. qRT-PCR analysis of *MsHHO3* in wild-type (WT) and *MsHHO3*-overexpressing lines. Student's t-test was used to identify significant differences (***P* < 0.01). (b) *pNifH:GUS* staining of nodules from WT and *MsHHO3*-overexpressing lines under N-deficient conditions. Scale bar=2 mm.

**Figure S8.**


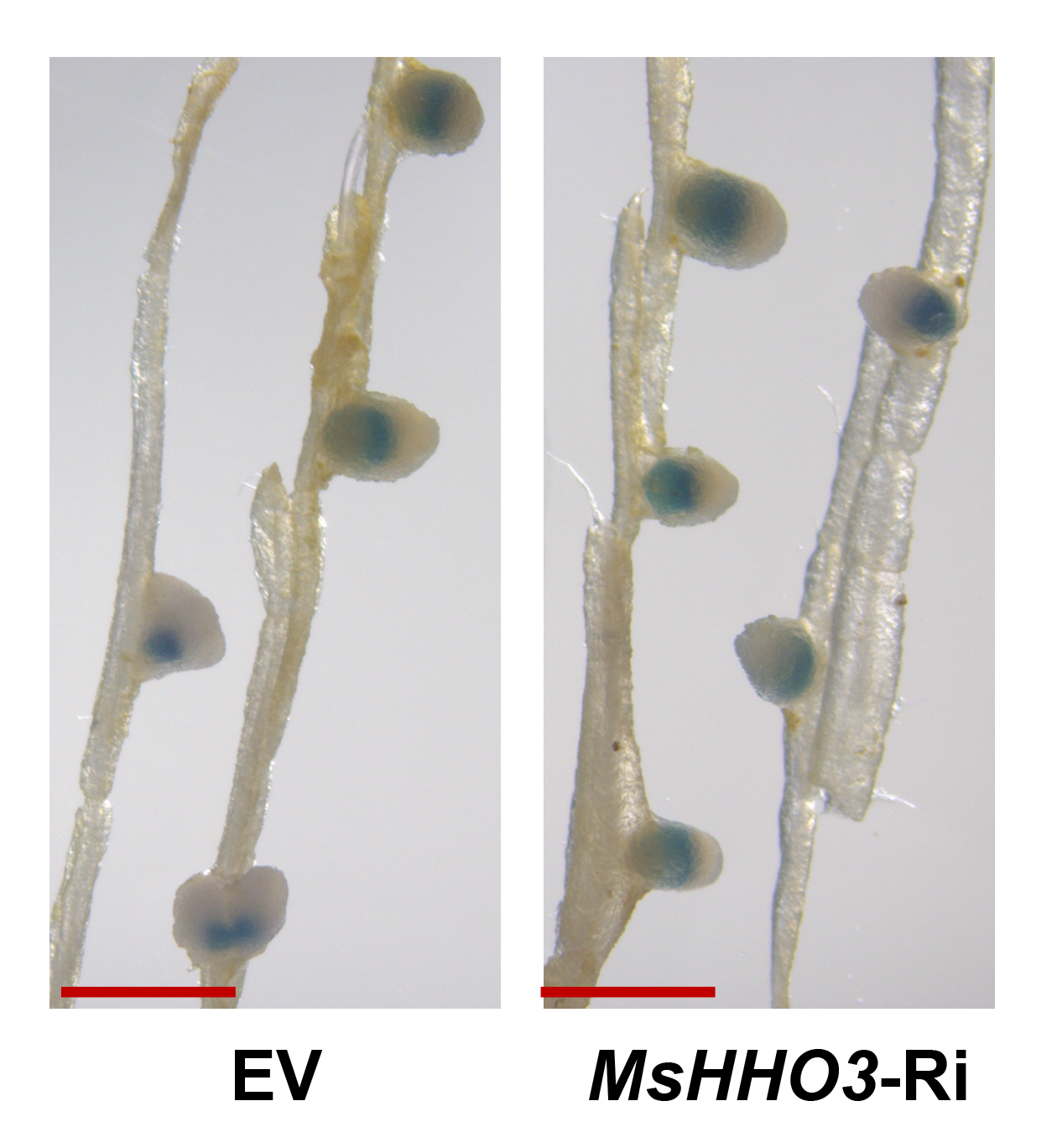


**Figure S8.** Nodules of EV and *MsHHO3*-Ri lines with *pNifH:GUS* staining under N-sufficient conditions. Scale bar=2 mm.

**Figure S9.**


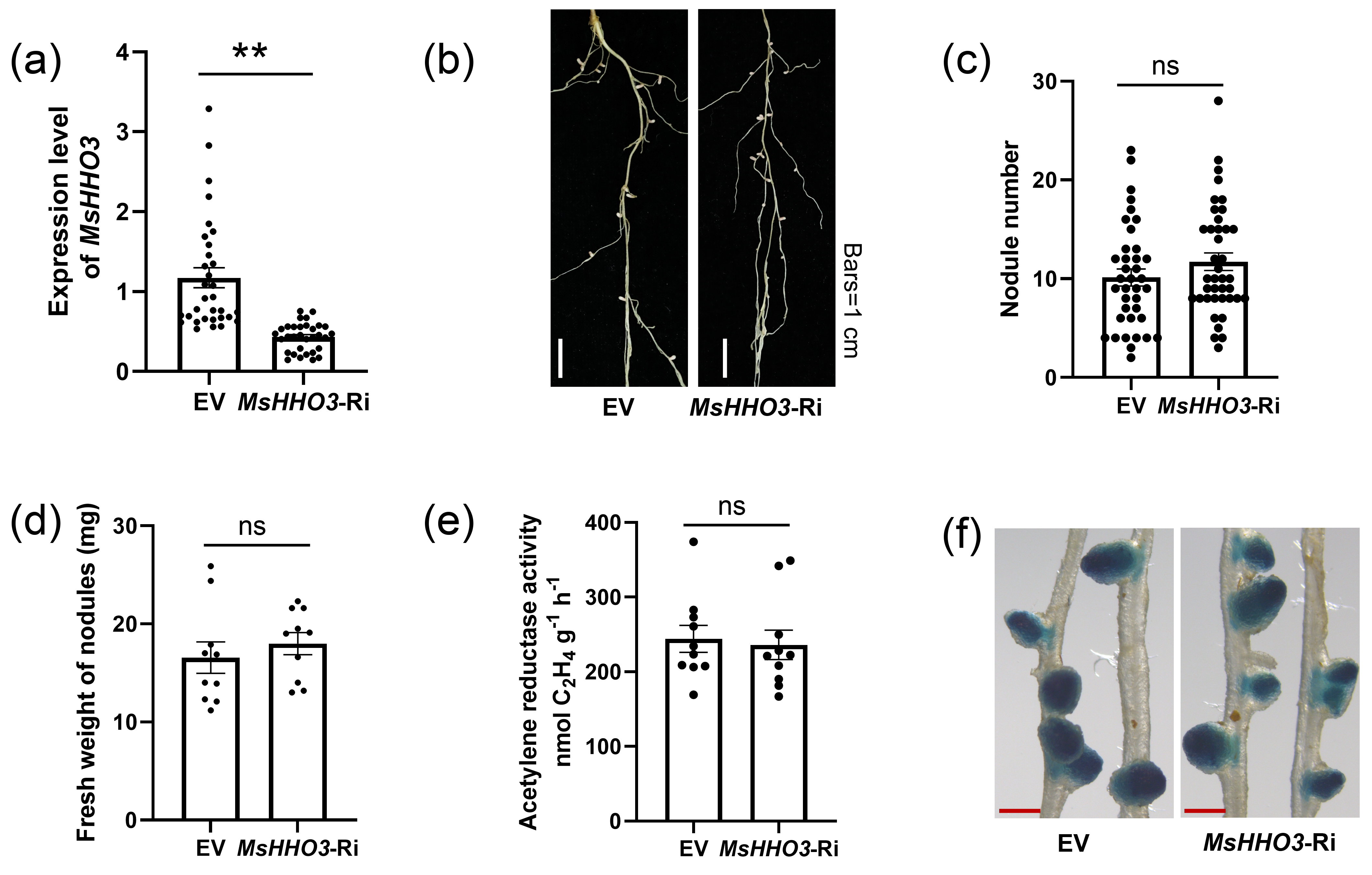


**Figure S9.** Phenotypic comparison of EV and *MsHHO3*-Ri under N-deficient conditions.

(a) qRT-PCR analysis of *MsHHO3* expression levels in hairy roots carrying the empty vector (EV) and *MsHHO3*-Ri vector. (b) Nodule phenotypes of *MsHHO3*-Ri and the EV. Scale bar=1 cm. (c) Total nodule number. (d) Fresh weight (FW) of nodules. (e) Nitrogenase activity. (f) Nodules of *pNifH:GUS* staining. Scale bar=1 mm. Two-tailed student's t-test was used to identify significant differences (***P* < 0.01).

**Figure S10.**


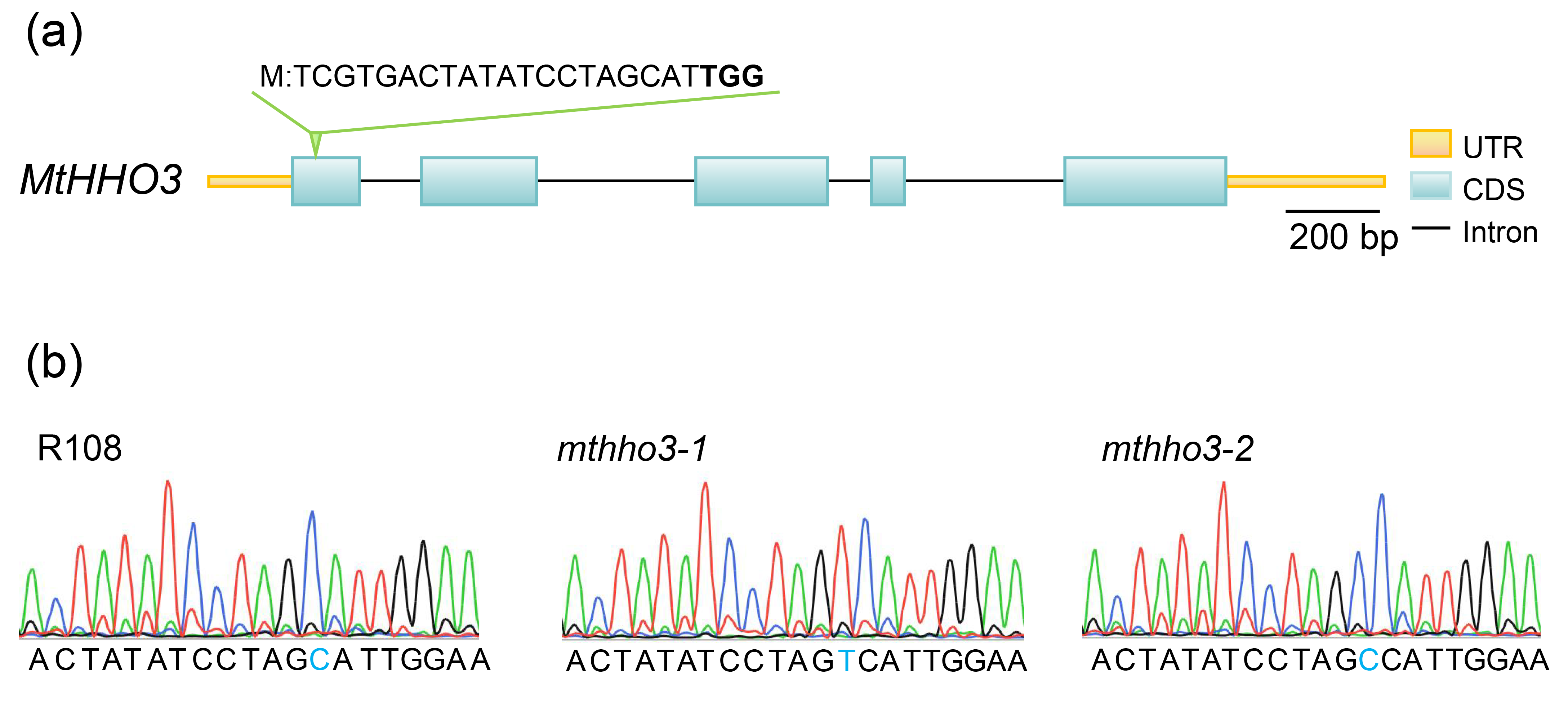


**Figure S10.** Construction of the *mthho3* mutant.

(a) Schematic diagram of the CRISPR/Cas9 target site (M) within the *MtHHO3*. The UTRs, exons and introns are represented by orange boxes, blue boxes and black lines, respectively. Protospacer adjacent motif (PAM) sequences are shown in bold black letters. (b) peak plots showing the CRISPR/Cas9 editing status of the *mthho3* mutant, with mutation sites marked by red letters.

**Figure S11.**


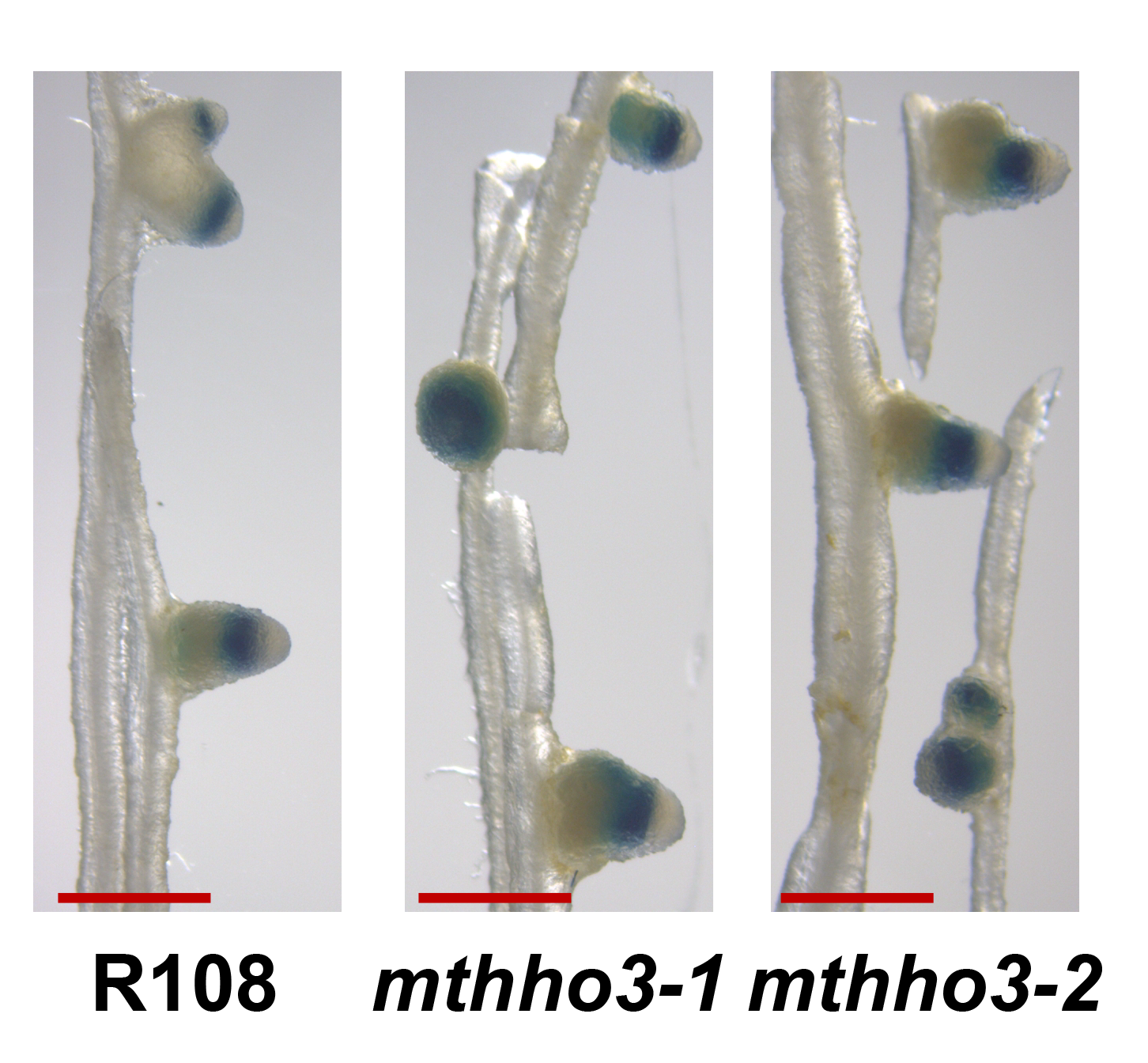


**Figure S11.** Nodules of wild type (R108) and *mthho3* mutants with *pNifH:GUS* staining under N-sufficient conditions. Scale bar=2 mm.

**Figure S12.**

**
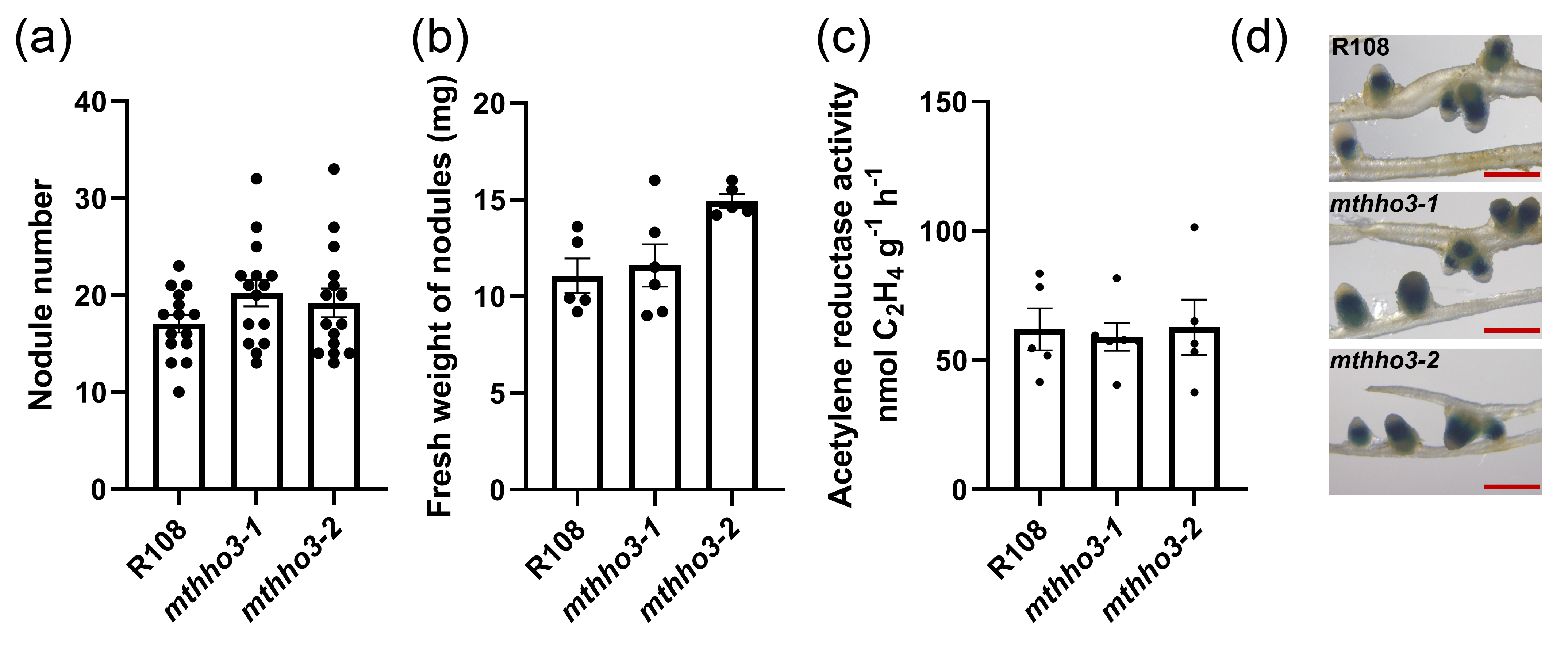
**

**Figure S12.** Nodule phenotypes of wild type (R108) and *mthho3* mutants under N-deficient conditions.

(a) Total nodule number. (b) Fresh weight (FW) of nodules. (c) Nitrogenase activity. (d) Nodules of *pNifH:GUS* staining. Scale bar=2 mm. Two-tailed student's t-test was used to identify significant difference.

**Figure S13.**


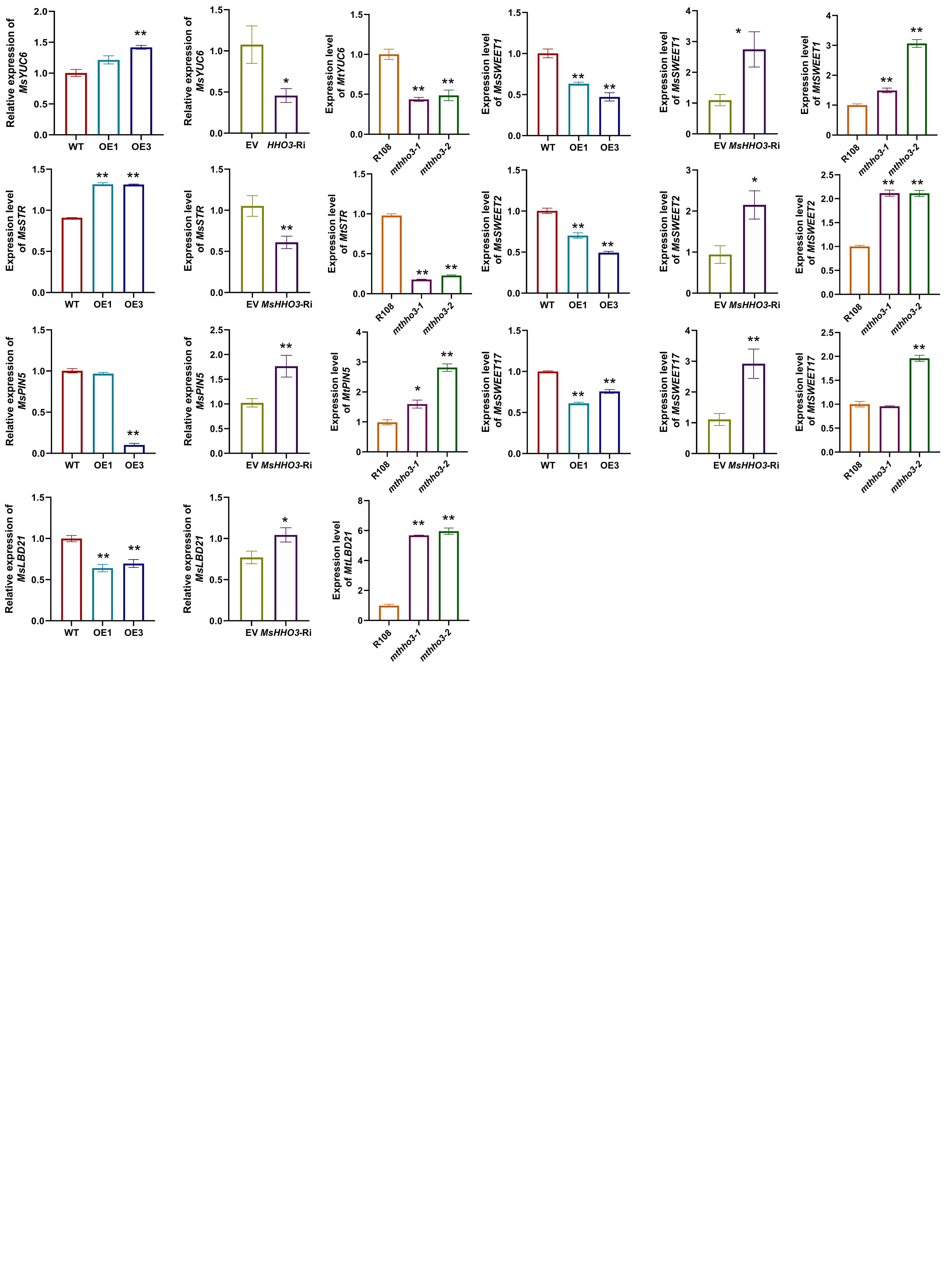


**Figure S13.** qRT-PCR analysis of the expression levels of nodulation-related genes identified by RNA-Seq in *MsHHO3*-overexpressing lines, *MsHHO3*-Ri and *mthho3* mutants compared to the control. Two-tailed student's t-test was used to identify significant difference.

**Figure S14.**


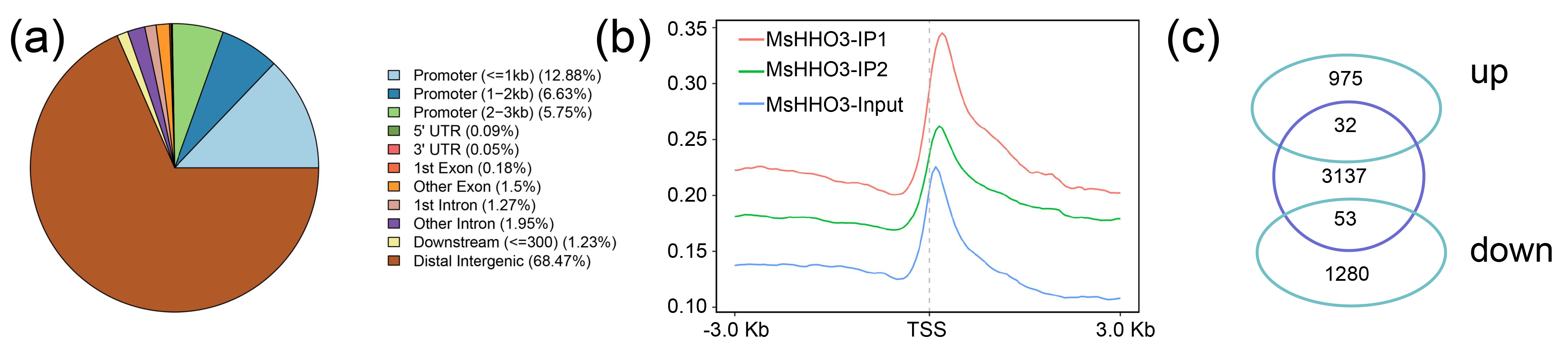


**Figure S14.** Integrated analysis of RNA-Seq and ChIP-Seq.

(a) Genomic distribution of MsHHO3 binding peaks. (b) ChIP-seq signal enrichment profile across a ±3 Kb window sounding the transcription start sites (TSS) of enriched genes. (c) Integrative analysis of RNA-seq and ChIP-seq data quantifies candidate direct targets with differential expression patterns. Blue and purple circles represent DEG numbers in RNA-seq and ChIP-seq data, respectively.

**Figure S15.**


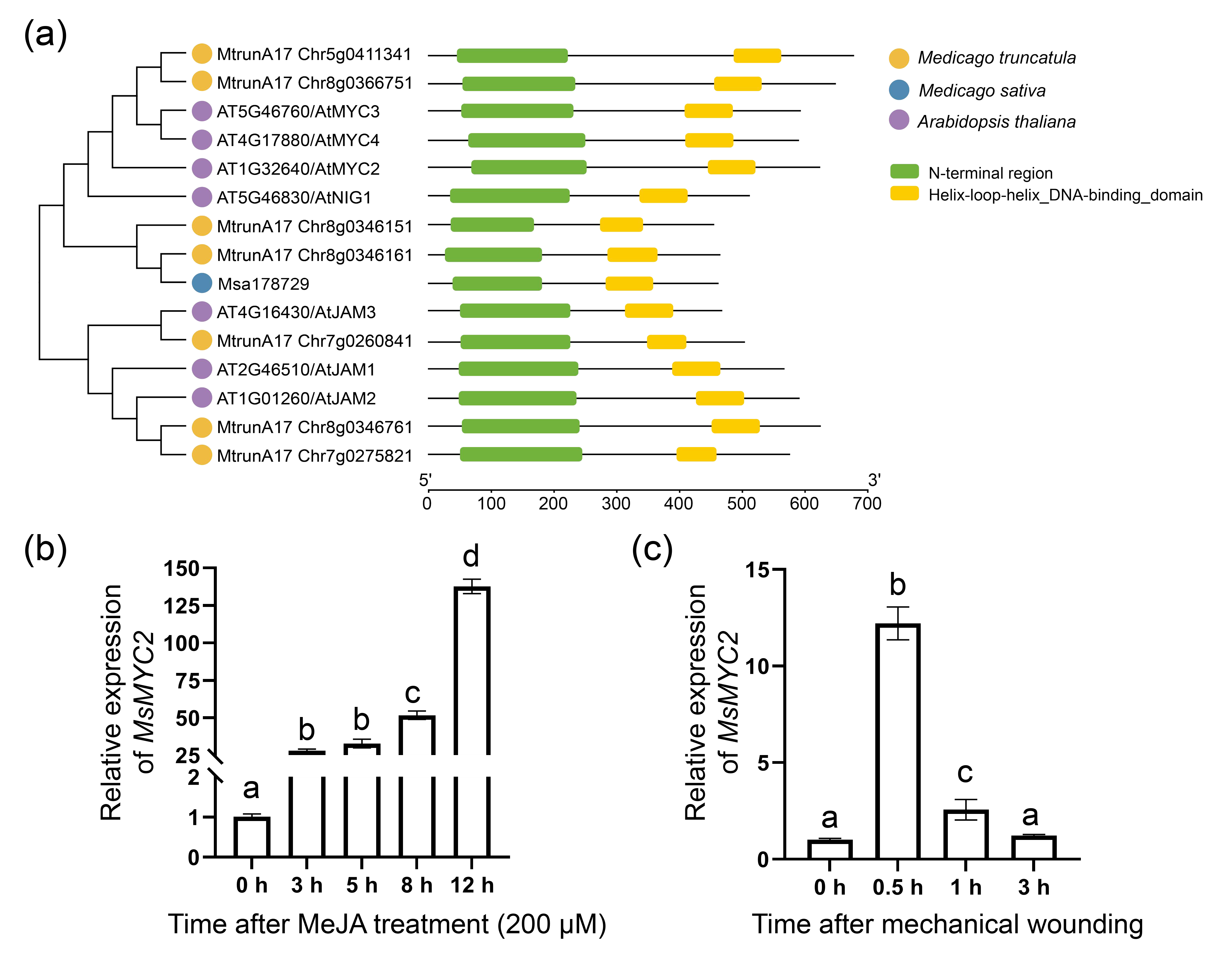


**Figure S15.** Characteristics of MsMYC2.

(a) Phylogenetic analysis of bHLH proteins. Species are distinguished by blue, yellow, and purple circles, respectively. (b) Expression analysis of *MsMYC2* after application of 200 μM MeJA or mechanical wounding. Statistical significance was determined by one-way analysis of variance (ANOVA) followed by Duncan’s multiple range test, as indicated by letters (*P*＜0.05).
